# *N*,*N*′‑Disubstituted Tetrandrine Derivatives: Enhanced Antibacterial Activity via Double Quaternization

**DOI:** 10.1021/acsomega.6c05294

**Published:** 2026-08-04

**Authors:** Viviana I. Calvillo-Páez, Adrián Ochoa-Terán, Juan Carlos Gálvez-Ruiz, Martin Samuel Hernández-Zazueta, Carmen L. Del-Toro-Sánchez, Luisa A. Rascón-Valenzuela, J. Octavio Juárez-Sánchez, Jancarlo Gomez-Vega, Maribel Plascencia-Jatomea, Karen Ochoa Lara

**Affiliations:** † Centro de Graduados e Investigación en Química, Tecnológico Nacional de México, Campus Tijuana, CP 22444 Tijuana, Baja California, Mexico; ‡ Departamento de Investigación en Polímeros y Materiales, 27813 Universidad de Sonora, Rosales y Encinas s/n, Col. Centro, CP 83000 Hermosillo, Sonora, Mexico; § Departamento de Ciencias Químico-Biológicas, Universidad de Sonora, Rosales y Encinas s/n, Col. Centro, CP 83000 Hermosillo, Sonora, Mexico; ∥ Departamento de Investigación y Posgrado en Alimentos, Universidad de Sonora, Rosales y Encinas s/n, Col. Centro, CP 83000 Hermosillo, Sonora, Mexico; ⊥ Departamento de Investigación en Física, Universidad de Sonora, Rosales y Encinas s/n, Col. Centro, CP 83000 Hermosillo, Sonora, Mexico

## Abstract

A series of *N*,*N*′-disubstituted tetrandrine derivatives, comprising both newly synthesized and previously reported compounds, were investigated as a strategy to enhance antibacterial activity. Four derivatives (**DIT**, **DAT**, **DNT**, and **DBT**) had been previously reported by our group, whereas three new analogues (**DQT**, **DAcT**, and **DeBT**) were synthesized and fully characterized. Antibacterial effects were evaluated by the broth microdilution method against *Escherichia coli*, *Staphylococcus aureus*, *Enterococcus faecalis*, and *Pseudomonas aeruginosa*. In contrast to natural tetrandrine, quaternized derivatives exhibited markedly enhanced antibacterial potency, particularly against *S. aureus*, highlighting **DAcT** (minimum inhibitory concentration (MIC) = 0.04 μg/mL), **DQT** (MIC = 0.11 μg/mL), and **DNT** (MIC = 0.14 μg/mL), with activities comparing favorably to those of reference antibiotics such as ampicillin, chloramphenicol, and ciprofloxacin. Antioxidant activity was also assessed using ABTS^•+^ and DPPH^•^ assays, with the highest response observed in the ABTS^•+^ assay for **DeBT** (63.72% inhibition, IC_50_ = 21.53 μg/mL), followed by **DNT** (40.97% inhibition). Cytotoxicity studies of the newly synthesized derivatives against ARPE-19, MCF-7, HeLa, and A549 cell lines revealed low toxicity (IC_50_ > 50 μg/mL), consistent with previously reported analogues, suggesting a favorable selectivity profile. DNA-binding studies by fluorescence spectroscopy indicated efficient interaction with double-stranded DNA. In addition, molecular modeling and docking studies against relevant bacterial targets provided mechanistic insight and showed good agreement with the experimental antibacterial results. Overall, the combined in vitro and in silico findings identify *N*,*N*′-disubstituted tetrandrine derivatives as promising scaffolds for the development of new antibacterial agents.

## Graphical abstract

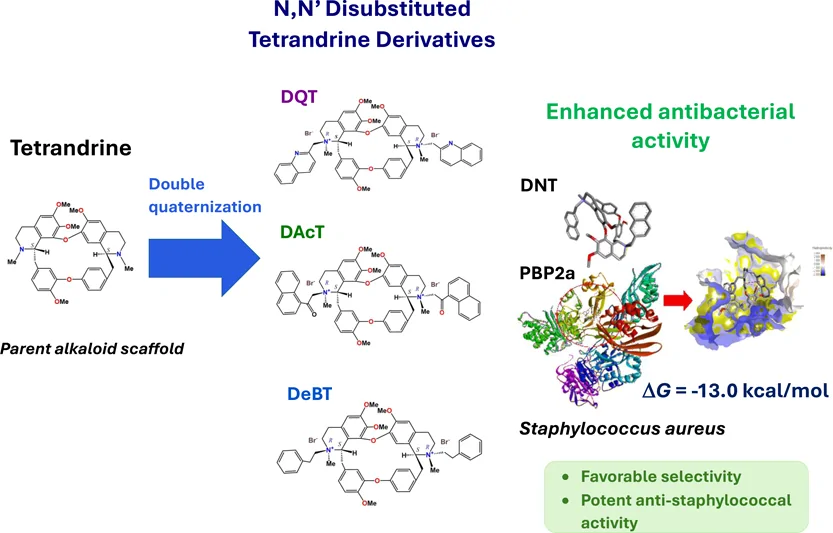

## 1 Introduction

The World Health Organization reports that antimicrobial resistance is rising to dangerously high levels worldwide and calls for coordinated measures to address this problem. Without urgent action, we risk entering a postantibiotic era in which common infections and minor injuries can once again be fatal.^1,2^ The Infectious Diseases Society of America (IDSA) highlights the ESKAPE pathogens (*Enterococcus faecium*, *Staphylococcus aureus*, *Klebsiella pneumoniae*, *Acinetobacter baumannii*, *Pseudomonas aeruginosa*, and *Enterobacter* spp.), which together account for roughly two-thirds of healthcare-associated infections.^3^

Since 2002, IDSA has underscored the limited progress in discovering new antibacterial agents to treat multidrug-resistant infections.^4,5^ In this context, there is a pressing need for compounds with greater potency, broader spectra of activity, and improved safety profiles. Consequently, researchers worldwide are pursuing alternatives that target novel mechanisms of action and therapeutic targets.^5^ Mechanisms under active investigation include double-stranded DNA (dsDNA) intercalation, membrane disruption, and the generation of reactive oxygen species, leading to bacterial cell death.^6,7^

For centuries, nature has provided bioactive compounds for the treatment of a broad range of diseases. Plants have been an important source of pharmaceutical agents.^8^ Among natural products, alkaloids are notable for their structural diversity and wide spectrum of biological activities. Since antiquity, plants rich in alkaloids have been used as poisons, stimulants, narcotics, and medicines.^9,10^

*S*,*S*-(+)-Tetrandrine is a bisbenzylisoquinoline alkaloid widely recognized for its broad range of biological properties, including anticancer, calcium channel-blocking, immunosuppressive, anti-inflammatory, antidiabetic, antioxidant, and antimicrobial activities.^11–13^ Furthermore, tetrandrine has also demonstrated antiviral activity against SARS-CoV-2.^14^

Regarding its antibacterial profile, tetrandrine generally exhibits poor intrinsic activity against *S. aureus* (minimum inhibitory concentration (MIC) ≥ 250 μg/mL) under standard broth microdilution conditions, although synergistic effects with conventional antibiotics have been reported, particularly against methicillin-resistant *S. aureus* (MRSA), as well as against *Mycobacterium* spp. and *Salmonella* spp.^15–17^

On the other hand, our research group has reported several studies on the synthesis of tetrandrine derivatives via quaternization of the nitrogen atoms, demonstrating that this strategy can improve the physicochemical and pharmacological properties of natural tetrandrine.^18–26^ This strategy has been recognized as a useful approach for improving the physicochemical and pharmacological properties of natural tetrandrine.^27^ In particular, a series of disubstituted tetrandrine derivatives showed high-affinity binding to several DNA models and low cytotoxicity,^25^ whereas two monosubstituted derivatives previously exhibited promising antibacterial activity, especially against *S. aureus*.^26^

Building on these findings, the present work describes the antibacterial evaluation of a series of *N*,*N*′-disubstituted tetrandrine derivatives (Figure 1), four of which were previously reported.^25^ Considering the previously reported antioxidant activity of tetrandrine, antioxidant activity (ABTS^•+^ and DPPH^•^ assays), was also evaluated as part of the biological evaluation of the new derivatives. Cytotoxicity in normal and tumor cell lines, dsDNA binding by the ethidium bromide displacement assay, and molecular modeling/docking studies against relevant bacterial targets were investigated. Overall, this study demonstrates that double quaternization is an effective strategy to enhance the antibacterial potential of the tetrandrine scaffold.

**Figure 1:**
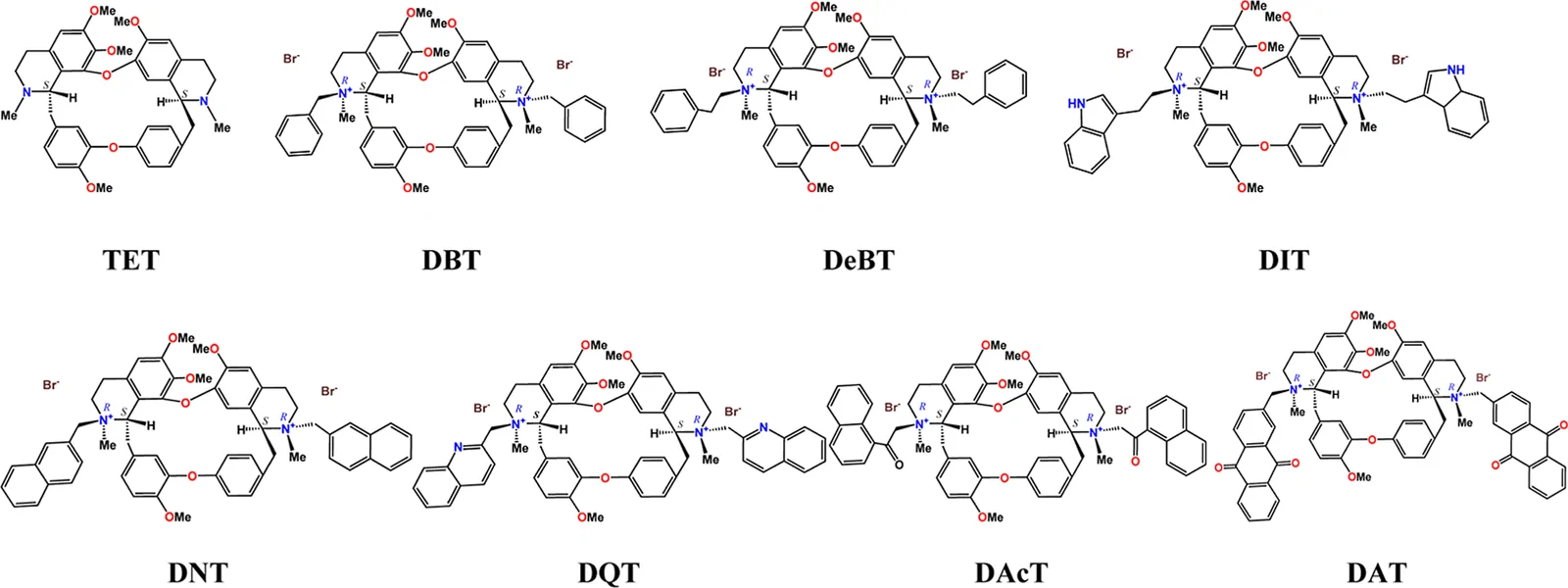
Chemical structures of tetrandrine and the disubstituted tetrandrine salts studied in this work. The chirality of the stereogenic centers in the isolated stereoisomers is indicated as *S* and *R*.

## 2 Results and Discussion

### 2.1 Synthesis and Structural Characterization

The *N*,*N*′-disubstituted derivatives **DBT**, **DNT**, **DAT**, and **DIT** were prepared in moderate to good yields, as previously reported in the literature.^18,21,24,25^ Similarly, the novel tetrandrine salts **DQT**, **DAcT**, and **DeBT** were synthesized by quaternization of the tertiary amine groups of the alkaloid with 2-(bromomethyl)­quinoline, 2-bromo-2′-acetonaphthone, and 2-bromoethylbenzene, respectively, in moderate to good yields.

Quaternization at the nitrogen atoms of *S*,*S*-(+)-tetrandrine generates two new stereogenic centers in the resulting derivatives. In all cases, a single stereoisomer was obtained, consistent with previous reports.^18,24,25^ The products were characterized by melting point, IR, NMR (^1^H and ^13^C) spectroscopy, using standard one- and two-dimensional techniques, as well as mass spectrometry and elemental analysis, providing data consistent with the proposed structures (see the Section 4).

According to the ^1^H NMR spectra, **DQT**, **DAcT**, and **DeBT** exist as single stereoisomers, showing a single set of signals for the hydrogen atoms. The ^1^H NMR and selected mass spectra of the new tetrandrine salts are provided in the Supporting Information (Figures S1–S6). Additionally, UV–vis absorption and emission spectra recorded in aqueous phosphate buffer (pH = 7.2) for each derivative are included in the Supporting Information (Figures S7 and S8). These results confirm the successful quaternization of the tetrandrine scaffold.

### 2.2 Antibacterial Activity

The antibacterial activity of derivatives **DBT**, **DeBT**, **DIT**, **DNT**, **DQT**, **DAcT**, and **DAT** was evaluated using the broth microdilution method against *S. aureus* ATCC 25923, *Escherichia coli* ATCC 25922, *E. faecalis* ATCC 29212, and *P. aeruginosa* ATCC 10145, as described in Section 4. MIC and half-maximal inhibitory concentration (IC_50_) values are summarized in Tables 1 and S1, respectively. As shown, natural tetrandrine did not exhibit antibacterial activity at the concentrations tested, whereas its derivatives displayed significant activity across the evaluated strains. In general, the derivatives showed higher activity against Gram-positive bacteria (*S. aureus* and *E. faecalis*) and moderate activity against Gram-negative bacteria (*E. coli* and *P. aeruginosa*), when compared with both tetrandrine and reference antibiotics. Notably, the most potent activity was observed against *S. aureus*, with **DAcT** (MIC = 0.04 μg/mL), **DQT** (MIC = 0.11 μg/mL), and **DNT** (MIC = 0.14 μg/mL), showing values comparable to or better than those of ampicillin (MIC = 9.08 μg/mL) and chloramphenicol (MIC = 1.19 μg/mL), and similar to ciprofloxacin (MIC = 0.04 μg/mL).^28^ Notably, several derivatives exhibited very high antibacterial activity, with MIC values below the lowest concentration tested, as indicated by values such as <0.20 μg/mL. This suggests that the true MIC values for these compounds may be significantly lower than those experimentally determined, highlighting their remarkable potency, particularly against Gram-positive strains. Additionally, **DQT** exhibited strong activity against *E. faecalis* (MIC < 0.20 μg/mL). Against *P. aeruginosa*, notable activity was observed for **DNT** (MIC = 0.90 μg/mL) and **DBT** (MIC = 0.89 μg/mL), outperforming chloramphenicol (MIC = 2.98 μg/mL). The only derivative that did not show measurable activity was **DAT**, likely due to MIC values above the highest concentration tested, similar to tetrandrine. Indeed, tetrandrine has been previously reported to exhibit MIC values greater than 250 μg/mL against *S. aureus* under comparable conditions,^15^ indicating that most derivatives significantly improve the antibacterial profile of the parent alkaloid. Importantly, previous antiproliferative studies using ARPE-19 cells demonstrated low cytotoxicity for **DBT** (IC_50_ > 30 μg/mL) and **DNT** (IC_50_ = 21.10 μg/mL),^25^ supporting their potential as promising antibacterial candidates with a favorable safety profile.

**Table 1:** MIC (μg/mL) of *N*,*N*′-Disubstituted Tetrandrine Derivatives and Antibiotics against Various Bacterial Strains^a^

	MIC (μg/mL ± SD)			
compound	S. aureus	E. faecalis	E. coli	P. aeruginosa
**DBT**	7.84 ± 0.42	3.05 ± 0.16	<1.07 ± 0.34	0.89 ± 0.10
**DeBT**	1.73 ± 0.22	4.80 ± 1.11	12.91 ± 2.29	1.10 ± 0.40
**DNT**	0.14 ± 0.03	<1.30 ± 0.35	ND*	0.90 ± 0.12
**DQT**	0.11 ± 0.04	<0.20 ± 0.17	13.32 ± 1.11	14.37 ± 1.26
**DAcT**	0.04 ± 0.02	ND*	3.64 ± 0.31	4.51 ± 0.56
**DIT**	7.05 ± 0.37	5.23 ± 0.43	ND*	ND*
**DAT**	NI*	NI*	ND*	ND*
**TET**	NI*	NI*	NI*	NI*
ampicillin	9.08 ± 0.92	0.006 ± 0.004	0.018 ± 0.007	65.45 ± 3.07
chloramphenicol	1.19 ± 0.15	49.34 ± 3.38	0.45 ± 0.06	2.98 ± 0.31

a Values are the mean ± standard deviation (SD) of three repetitions (*n* = 3). ND*: Not determined due to lack of fit to the Probit model. NI*: No inhibition was observed at the tested concentrations. Values preceded by “<” indicate MIC values below the lowest concentration tested.

Resazurin-based viability assays (Figure S9) further support these findings. In this method, the reduction of resazurin to resorufin serves as an indicator of bacterial viability, where blue wells indicate a loss of viability, and pink wells indicate active metabolism.^29,30^ The representative plates show consistent trends across all strains, with *S. aureus* and *E. faecalis* exhibiting the greatest susceptibility, followed by *P. aeruginosa*, as evidenced by an increased number of nonviable wells at higher concentrations.

A qualitative structure–activity analysis suggests that the nature of the substituents introduced at the nitrogen atoms plays an important role in modulating antibacterial activity. In particular, derivatives bearing extended fused aromatic or heteroaromatic substituents, including naphthyl, acetonaphthyl, and quinolyl groups such as **DAcT**, **DQT**, and **DNT** exhibited the highest activity, especially against *S. aureus*, which may be associated with increased lipophilicity and enhanced interactions with bacterial membranes or intracellular targets (e.g., dsDNA). In contrast, some derivatives such as **DAT** showed activity that could not be reliably quantified under the experimental conditions, likely due to limitations in model fitting (ND* values), rather than a complete lack of the antibacterial effect. Although these observations provide useful insight, further studies are required to establish definitive structure–activity relationships (SAR).

Overall, the enhanced antibacterial activity observed for the quaternized derivatives, particularly against Gram-positive bacteria, suggests that structural modification of tetrandrine plays a key role in modulating its biological activity. While the precise mechanism of action remains to be fully elucidated, these results are consistent with previously reported interactions of cationic compounds with bacterial membranes and intracellular targets.^31^ Further studies, including dsDNA binding and molecular docking analyses, are discussed in the following sections.

### 2.3 Antioxidant Activity

A previous study on mono-*N*-substituted tetrandrine derivatives demonstrated measurable antioxidant activity, particularly in the ABTS^•+^ assay.^26^ Therefore, the antioxidant activity of the *N*,*N*′-disubstituted derivatives was evaluated as part of their biological evaluation using the ABTS^•+^ and DPPH^•^ radical scavenging assays (see Section 4), with gallic acid as a positive control. As summarized in Table 2, the inhibition percentages for the DPPH^•^ radical were generally low for all compounds. This behavior may be attributed to the steric hindrance of the DPPH^•^ radical, whose reactive center is relatively shielded, making it less accessible to bulky molecules such as tetrandrine.^32^ Additionally, the polarity of the compounds influences their radical scavenging capacity, as DPPH^•^ is more selective compared to ABTS^•+^, which can react with both hydrophilic and lipophilic antioxidants.^33^ In contrast, higher inhibition values were observed for the ABTS^•+^ radical. Notably, tetrandrine exhibited the highest activity (90.92%), followed by **DeBT** (63.72%) and **DNT** (40.97%). Among the derivatives, only **DeBT** showed inhibition above 50%, allowing the determination of its IC_50_ value (21.53 μg/mL); see Table 3. This result is comparable to that reported for other alkaloids such as berberine (IC_50_ = 38.7 μg/mL) under similar conditions.^34^

**Table 2:** Effect of Tetrandrine (**TET**) and Its Derivatives on the Percentage of Inhibition of DPPH^•^ and ABTS^•+^ Free Radicals

	free radical inhibition percentages (%) ± SD^a^								
	**TET**	**DBT**	**DeBT**	**DIT**	**DNT**	**DQT**	**DAcT**	**DAT**	gallic acid
DPPH^•^	3.48 ± 1.46	NI**	NI**	4.97 ± 1.53	4.45 ± 1.27	6.75 ± 3.13	3.16 ± 0.69	NI**	93.34 ± 1.56
ABTS^•+^	90.92 ± 1.20	NI**	63.72 ± 1.70	22.09 ± 5.41	40.97 ± 6.92	20.67 ± 5.40	22.17 ± 2.72	2.40 ± 0.65	99.87 ± 1.32

a Data are expressed as mean ± SD of at least three independent experiments (*n* = 3). It was determined at a concentration of the compounds of 30 μg/mL; NI** = inhibition percentage was not obtained at the maximum concentration used.

**Table 3:** Half-Maximal Inhibitory Concentration IC_50_ (μg/mL)

	IC_50_ ± SD^a^		
	**TET**	**DeBT**	gallic acid
ABTS^•+^	4.63 ± 0.24	21.53 ± 0.28	2.53 ± 0.78

a Data are expressed as mean ± SD of at least three independent experiments (*n* = 3).

Overall, the antioxidant activity of the tetrandrine derivatives can be considered to be moderate and dependent on the nature of the substituents. Although antibacterial activity constitutes the primary focus of the present study, the antioxidant assays provide complementary information regarding the biological profiles of these compounds.

### 2.4 Cytotoxicity Evaluation and Biological Selectivity

The cytotoxicity of the newly synthesized tetrandrine derivatives (**DAcT**, **DQT**, and **DeBT**) was evaluated against three human cancer cell lines (MCF-7, HeLa, and A549) and a nontumoral cell line (ARPE-19). As summarized in Table 4, all compounds exhibited low cytotoxicity, with IC_50_ values predominantly above 50 μg/mL across all tested cell lines, in contrast to natural tetrandrine, which showed significantly higher antiproliferative activity under comparable conditions. This behavior is consistent with previous reports on bis-*N*-substituted tetrandrine derivatives,^25^ where cytotoxicity was found to depend strongly on the nature of the aromatic substituents. In particular, derivatives bearing extended polyaromatic systems (e.g., pyrenyl groups) display enhanced cytotoxicity, likely associated with more efficient dsDNA intercalation. Notably, such highly intercalating substituents were deliberately excluded in the present study to modulate biological activity and reduce undesired cytotoxic effects. The results obtained herein reveal a clear decoupling between dsDNA-binding capability and cytotoxicity, suggesting that the newly designed derivatives retain the ability to interact with biomolecular targets without inducing significant cellular damage. This observation may be rationalized in terms of a nondisruptive interaction mode, where binding to dsDNA or other intracellular components occurs without strong interference in critical biological processes.

**Table 4:** Cytotoxicity (IC_50_, μg/mL) of Newly Synthesized Tetrandrine Derivatives in Human Cell Lines

	cell lines (IC_50_ ± SD^a^, μg/mL)			
compound	ARPE-19	MCF-7	HeLa	A549
**TET**	15.61 ± 1.30	19.46 ± 1.97	28.54 ± 1.16	19.21 ± 1.84
**DeBT**	85.37 ± 2.39	>200	119.60 ± 2.00	159.30 ± 5.13
**DQT**	78.82 ± 2.63	>200	90.62 ± 2.28	94.65 ± 1.69
**DAcT**	70.00 ± 1.33	53.26 ± 3.06	49.63 ± 0.65	57.84 ± 2.82

a Data are expressed as mean ± SD of at least three independent experiments (*n* = 3). ARPE-19: human retinal pigment epithelial cells (nontumoral); MCF-7: human breast adenocarcinoma; HeLa: human cervical carcinoma; A549: human lung carcinoma. Values reported as >200 indicate that 50% inhibition was not reached at the highest concentration tested.

Importantly, when these findings are considered in conjunction with the significant antibacterial activity described above, a coherent biological profile emerges. The most active derivatives (e.g., **DAcT**, **DQT**, and **DNT**), which exhibited potent antibacterial effects, particularly against Gram-positive strains, also displayed low cytotoxicity toward mammalian cells, indicating a favorable selectivity window. This balance between efficacy and limited host cell toxicity is a desirable feature in the development of new antimicrobial agents, particularly in the context of increasing bacterial resistance.^35^

From a pharmacological perspective, this combined behavior suggests that the antibacterial activity of these compounds is not simply a consequence of general cytotoxic effects but rather may arise from more selective interactions with bacterial targets. Therefore, the tetrandrine derivatives reported herein can be regarded as promising selective antibacterial agents with reduced potential for off-target effects in mammalian systems.

### 2.5 dsDNA-Binding Studies

Considering that many bioactive compounds, including antibiotics and anticancer agents, interact with dsDNA as part of their mechanism of action through intercalative or groove-binding modes,^36^ it was important to evaluate the DNA binding capacity of the tetrandrine derivatives reported herein.

Thus, the dsDNA-binding properties of **DQT**, **DAcT**, and **DeBT** were investigated using the ethidium bromide (EtBr) displacement assay. For comparison, previously reported data for **TET**, **DNT**, **DIT**, and **DAT** were also considered.^25^ The studies were performed by monitoring fluorescence changes of the EtBr–dsDNA complex ([DNA] = 10 μM, [EtBr] = 5 μM), following established methodologies.^23,25^

Representative spectra for **DQT** studies are shown in Figure 2, while titrations for **DAcT** and **DeBT** are provided in the Supporting Information (Figures S10 and S11). In all cases, the EtBr–dsDNA complex exhibited intense fluorescence emission at 600 nm upon excitation at 510 nm. Upon incremental addition of **DQT** (0–25 μM), a progressive decrease in fluorescence intensity was observed, reaching 43.3% quenching. Similarly, **DAcT** and **DeBT** induced fluorescence quenching of 42.1 and 31.1%, respectively.

**Figure 2:**
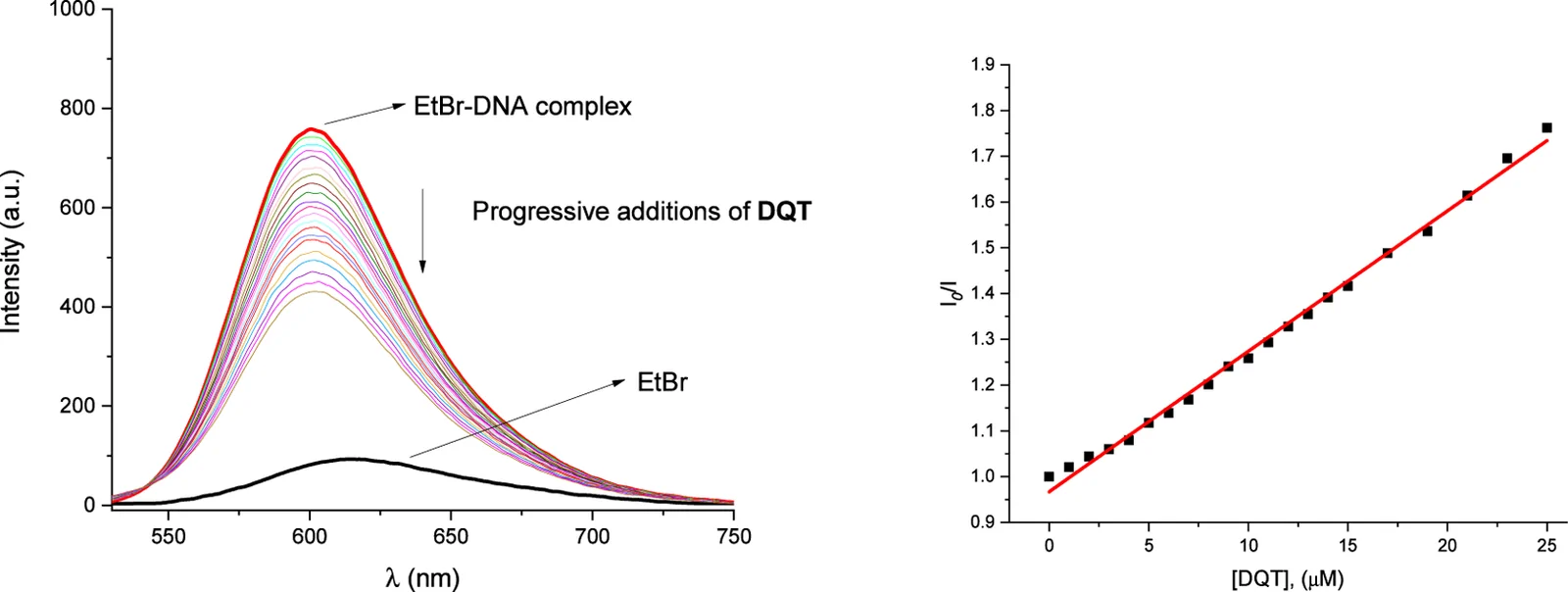
Emission spectra and fitting curve of the EtBr–DNA system in the presence of **DQT**, λ_exc_ = 510 nm, in phosphate buffer pH = 7.2, 0.01 M NaCl, and 10% dimethyl sulfoxide (DMSO) (v/v), at 25 °C. [EtBr] = 5 μM, [DNA] = 10 μM, [**DQT**] = 0–25 μM. ds-DNA: 5′-GTA AGA TGA TTC-3′ and 5′-GAA TCA TCT TAC-3′.

The Stern–Volmer quenching constants (*K*_sv_) for tetrandrine and its derivatives are summarized in Table 5. Notably, the derivatives generally exhibited higher *K*_sv_ values compared to natural tetrandrine, indicating an enhanced ability to interact with dsDNA. Among the compounds evaluated, **DAT** displayed the highest *K*_sv_ value, suggesting a stronger interaction with DNA relative to the other derivatives.

**Table 5:** Quenching Constants (*K*_*sv*_) for Complexes of Tetrandrine and Derivatives with ds-DNA^a^, in Phosphate Buffer pH = 7.2, 0.01 M NaCl, and 10% DMSO (v/v), at 25 °C

compound	*K*_sv_
**TET** ^b^	7.34 × 10^3^
**DeBT**	1.96 × 10^4^
**DNT** ^b^	1.69 × 10^4^
**DQT**	2.96 × 10^4^
**DAcT**	2.51 × 10^4^
**DAT** ^b^	1.33 × 10^5^
**DIT** ^b^	2.72 × 10^4^

a DNA: 5′-GTA AGA TGA TTC-3′ and 5′-GAA TCA TCT TAC-3′.

b Previously reported.^25^

These results indicate that *N*,*N*′-disubstituted tetrandrine derivatives have the ability to interact with dsDNA, likely through competitive displacement of EtBr. However, it is important to note that EtBr displacement assays alone do not allow unambiguous differentiation between intercalative and groove-binding modes, and therefore, the observed behavior should be interpreted as indicative of DNA interaction rather than definitive proof of a specific binding mechanism.^37^ Importantly, when considered alongside the cytotoxicity results, the observed DNA-binding capability does not correlate directly with increased antiproliferative effects. This suggests that these derivatives may interact with DNA in a nondisruptive manner, which is consistent with the low cytotoxicity observed in mammalian cells. Overall, the combination of dsDNA-binding ability, low cytotoxicity, and strong antibacterial activity supports the hypothesis that these compounds may exert their biological effects through selective interactions with bacterial targets, rather than through general cytotoxic mechanisms.

### 2.6 Molecular Modeling and Docking Studies

Prior to docking calculations, the molecular structures of the newly synthesized derivatives **DQT** and **DAcT** were constructed using crystallographically characterized tetrandrine analogues **DNT** and **DBT** previously reported by our group, whose solid-state structures were established by single-crystal X-ray diffraction.^24,25^ These compounds provide reliable structural templates for preserving the experimentally validated tetrandrine framework and stereochemical arrangement around the newly generated quaternary nitrogen centers. Subsequently, the structures of **DQT** and **DAcT** were geometry-optimized by density functional theory (DFT) calculations (see Section 4), affording energetically minimized three-dimensional models suitable for docking studies.

To gain insight into the possible antibacterial mechanisms of the tetrandrine derivatives, molecular docking studies were carried out against selected bacterial targets relevant to the strains evaluated experimentally, including TEM-1 β-lactamase (*E. coli*), AmpC β-lactamase (*P. aeruginosa*), penicillin-binding proteins (PBP4 and PBP2a), and chloramphenicol acetyltransferase III (CAT III) from Gram-positive bacteria. These proteins are recognized targets associated with bacterial resistance, cell wall biosynthesis, and antibiotic inactivation and have been explored in structure-based antibacterial drug discovery studies.^38–40^ Representative results are summarized in Table 6, whereas additional docking data, including those for tetrandrine (**TET**), additional derivatives, reference drugs, and complementary interaction figures, are provided in the Supporting Information (Table S2, Figures S12 and S13).

**Table 6:** Representative Molecular Docking Results of Tetrandrine Derivatives against Selected Bacterial Targets and Correlation with In Vitro Antibacterial Activity

strain	compound	MIC (μg/mL)^a^	target	Δ*G* (kcal/mol)^b^
S. aureus	**DNT**	0.14	PBP2a	–13.0
S. aureus	**DAcT**	0.04	CAT III	–10.1
E. faecalis	**DQT**	<0.20	PBP4	–10.5
E. coli	**DBT**	<1.07	TEM-1	–7.7
P. aeruginosa	**DBT**	0.89	AmpC	–8.4

a MIC values correspond to minimum inhibitory concentrations determined experimentally.

b Predicted binding free energies (Δ*G*, kcal/mol) were obtained by molecular docking calculations. Lower (more negative) values indicate stronger predicted affinity. Complete docking results, including tetrandrine (**TET**), additional derivatives, commercial reference drugs, and detailed interaction data, are provided in the Supporting Information.

Overall, the predicted binding affinities showed clear agreement with the in vitro antibacterial activity results. In general, the most active derivatives against a given bacterial strain also displayed favorable calculated binding energies toward proteins associated with that microorganism, supporting the relevance of the selected targets.

In the case of Gram-negative bacteria, **DBT** showed favorable interactions with TEM-1 β-lactamase (Δ*G* = −7.7 kcal/mol) and AmpC β-lactamase (Δ*G* = −8.4 kcal/mol), as shown in Figure 3, in agreement with its notable antibacterial activity against *E. coli* and *P. aeruginosa*. These results suggest that interference with resistance-associated β-lactamase enzymes may contribute, at least in part, to the antibacterial profile of this derivative.^41^

**Figure 3:**
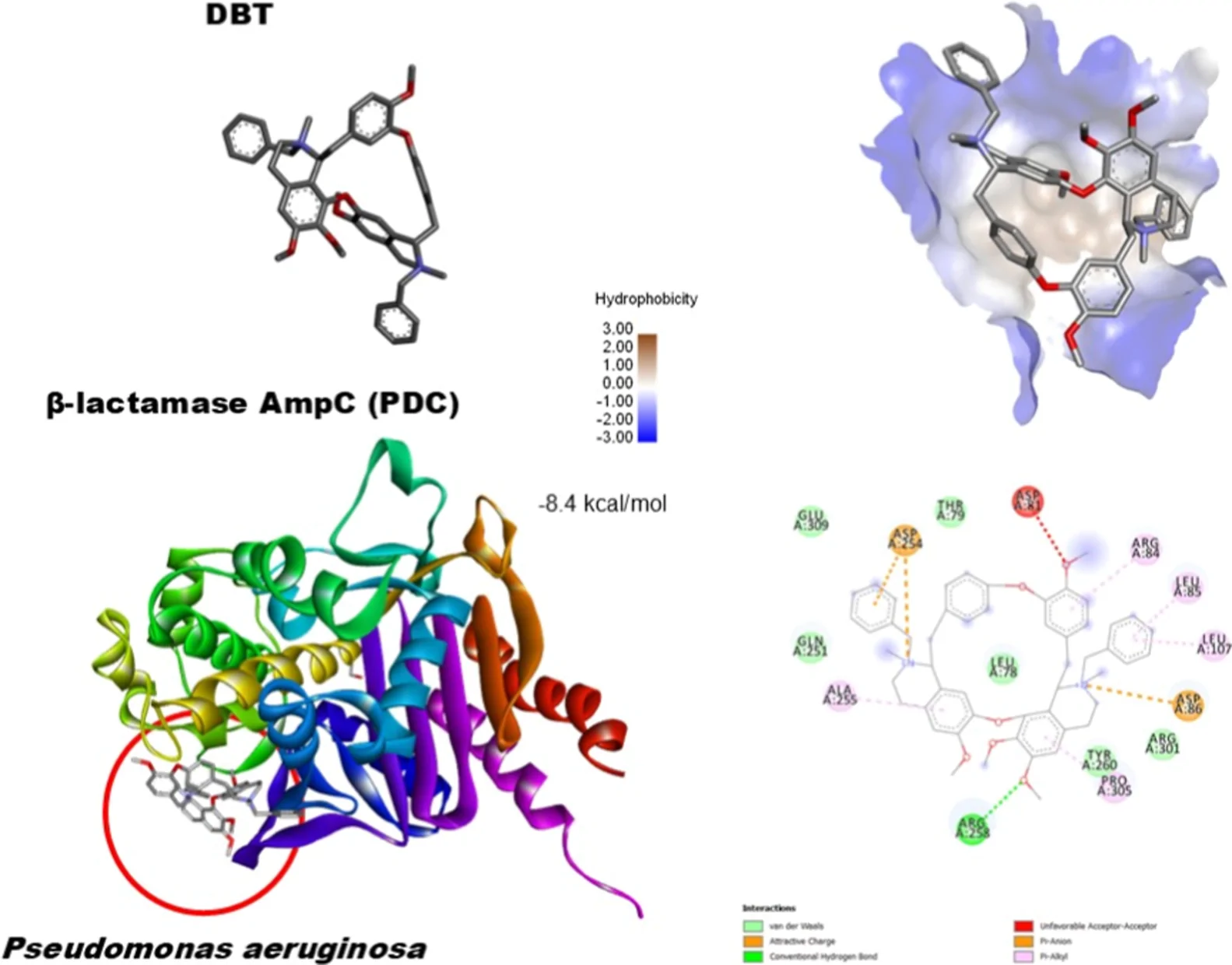
Predicted binding mode of **DBT** within AmpC β-lactamase (PDC) from *P. aeruginosa*. Composite representation of the **DBT**–AmpC complex showing: (upper panels) ligand accommodation within the hydrophobic binding pocket; (lower-left) overall protein cartoon with the active-site region highlighted; and (lower-right) 2D interaction map with key contacting residues. The favorable predicted binding affinity (Δ*G* = −8.4 kcal/mol) is consistent with the notable antibacterial activity of **DBT** against *P. aeruginosa* observed experimentally. Residue numbering follows PDB chain labels.

For Gram-positive strains, highly favorable interactions were predicted for **DQT** with PBP4 from *E. faecalis* (Δ*G* = −10.5 kcal/mol), consistent with its remarkable antibacterial activity against this strain. Likewise, **DAcT** displayed strong predicted affinity toward CAT III from *S. aureus* (Δ*G* = −10.1 kcal/mol), correlating with its potent antistaphylococcal activity. Particularly noteworthy was **DNT**, which showed excellent predicted binding toward PBP2a from *S. aureus* (Δ*G* = −13.0 kcal/mol), Figure 4, together with strong in vitro activity (MIC = 0.14 μg/mL). Since PBP2a is a clinically relevant transpeptidase associated with resistance to β-lactam antibiotics, this result suggests that interactions with cell wall biosynthesis proteins may contribute to the pronounced antistaphylococcal activity of this derivative.^40,42^

**Figure 4:**
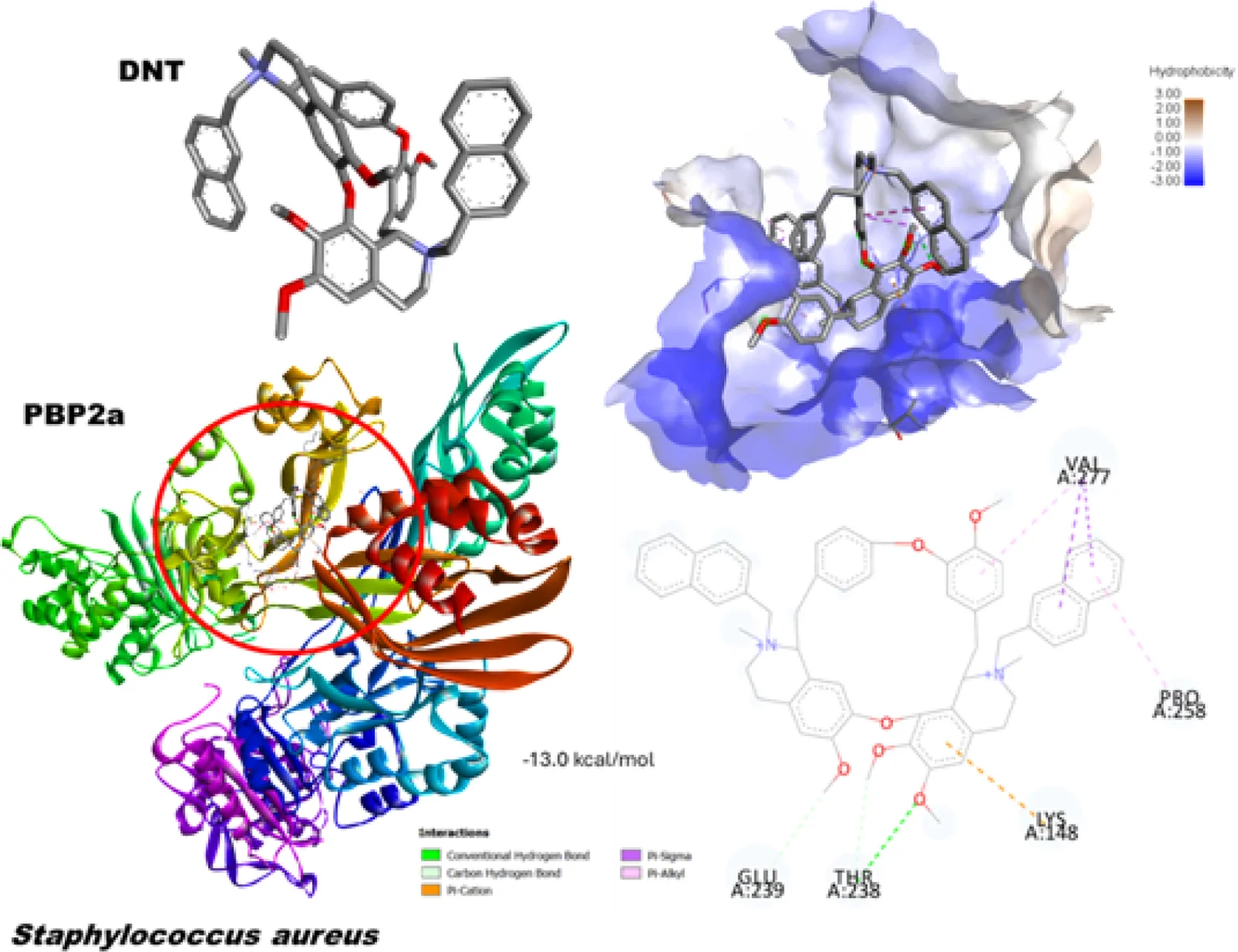
Predicted binding mode of **DNT** within the PBP2a active site from *S. aureus*. Composite representation of the **DNT**–PBP2a complex showing: (upper panels) ligand accommodation within the hydrophobic protein surface pocket; (lower-left) overall protein cartoon with the active-site cavity highlighted; and (lower-right) 2D interaction map with key contacting residues. The favorable predicted binding affinity (Δ*G* = −13.0 kcal/mol) is consistent with the strong antistaphylococcal activity of **DNT** observed experimentally. Residue numbering follows PDB chain labels.

Natural tetrandrine also showed measurable affinity toward some of the evaluated proteins; however, overall, the quaternized *N*,*N*′-disubstituted derivatives exhibited stronger predicted interactions and markedly enhanced antibacterial activity relative to the parent alkaloid. This trend is consistent with the beneficial effect of cationic functionalization discussed throughout this work.^28^

Taken together, the docking results complement the biological assays, cytotoxicity studies, and dsDNA-binding experiments, reinforcing the hypothesis that rationally modified tetrandrine derivatives can act as selective antibacterial agents through favorable interactions with relevant bacterial targets. Although molecular docking provides only a theoretical approximation of binding, the strong consistency observed with the experimental data supports the proposed structure–activity relationships.^43^

## 3 Conclusions

In this work, a focused library of *N*,*N*′-disubstituted tetrandrine derivatives, including three newly synthesized compounds (**DeBT**, **DQT**, and **DAcT**), was successfully prepared and comprehensively evaluated as potential antibacterial agents. Structural modification of the tetrandrine scaffold through double quaternization proved to be an effective strategy to markedly enhance the biological activity of the parent alkaloid.

Several derivatives exhibited outstanding antibacterial potency, particularly against Gram-positive bacteria. Notably, **DAcT**, **DQT**, and **DNT** showed remarkable activity against *S. aureus*, with MIC values of 0.04, 0.11, and 0.14 μg/mL, respectively, comparing favorably with reference antibiotics. In addition, **DQT** displayed excellent activity against *E. faecalis*, while **DBT** showed relevant activity against Gram-negative strains such as *E. coli* and *P. aeruginosa*.

Importantly, the newly synthesized derivatives displayed low cytotoxicity toward mammalian cell lines, supporting a favorable selectivity profile. DNA-binding studies confirmed that these compounds retain the ability to interact with dsDNA, whereas molecular modeling and docking studies showed a clear correlation between the predicted target affinity and experimental antibacterial activity. In particular, favorable interactions with clinically relevant proteins such as PBP2a, PBP4, TEM-1, and AmpC provide plausible mechanistic support for the observed bioactivity.

Taken together, these results identify *N*,*N*′-disubstituted tetrandrine derivatives as promising antibacterial scaffolds for further development. The combination of potent antibacterial activity, low cytotoxicity, and tunable molecular architecture highlights the value of semisynthetic alkaloid functionalization as a strategy for the development of new antibacterial agents.

Current efforts in our laboratory are focused on the preparation of expanded derivative libraries with systematic structural variations based on the most active compounds identified herein, together with an evaluation against additional clinically relevant bacterial strains, resistance phenotypes, and preliminary in vivo studies.

## 4 Experimental Section

### 4.1 Reagents, Synthesis, and Characterization

All reagents and solvents were obtained from commercial suppliers and used as received without further purification. The previously reported tetrandrine derivatives **DBT**, **DNT**, **DAT**, and **DIT** were prepared according to the literature procedures.^18,21,24,25^ The synthetic procedures for new derivatives **DQT**, **DAcT**, and **DeBT** are described below.

### 4.2 Instrumentation

NMR spectra were recorded on a Bruker Avance III 400 spectrometer. Decomposition points were determined using a Büchi B-545 melting point apparatus. Infrared spectra were obtained on a PerkinElmer FRONTIER Fourier Transform Infrared spectrophotometer using KBr pellets. Mass spectra were acquired on an Agilent Technologies 6100 LC/MS system operating in positive electrospray ionization (ESI^+^) and atmospheric pressure chemical ionization (APCI^+^) modes. UV–vis absorption and fluorescence spectra were recorded using an Agilent Technologies 8435 diode-array spectrophotometer and a PerkinElmer LS50B fluorometer, respectively. Optical density values for antibacterial assays were measured using a BioTek 800 TS microplate reader. Absorbance measurements for antioxidant assays were performed in a 96-well Thermo Fisher Scientific microplate reader.

### 4.3 General Procedure for Double Quaternization of Tetrandrine

A solution of tetrandrine (200 mg, 0.29 mmol) and the corresponding alkyl bromide (2.2–3.0 equiv) in acetone (50–60 mL) was stirred under reflux, and the reaction progress was monitored by thin layer chromatography. After completion, the precipitated solid was collected by filtration or, when necessary, the solvent was removed under reduced pressure and precipitation was induced by addition of ethyl acetate. The crude product was washed with ethyl acetate and/or acetone and recrystallized from water/acetone to afford the corresponding bis-quaternary tetrandrine derivative.

#### 4.3.1 Synthesis of **DQT**

This derivative was prepared according to the general procedure using 2-(bromomethyl)­quinoline (146 mg, 0.65 mmol) under reflux for 24 h. Recrystallization from water/acetone afforded the product as a pale cream powder (230 mg, 74%). Decomposition point: 186.0–189.0 °C. UV–vis (DMSO/phosphate buffer, 1:9 v/v), λ_max_ (nm), ε (dm^3^ mol^–1^ cm^–1^): 320, 5164; 282, 11,389. ^1^H NMR (400 MHz, DMSO-*d*_6_) partial assignment was supported by two-dimensional techniques. Signals for –N^+^–CH_3_ and –O–CH_3_ were found at δ = 2.89 (s, 3H), 3.00 (s, 3H), 3.12 (s, 3H), 3.43 (s, 3H), 3.75 (s, 3H) and 3.78 (s, 3H). Signals for –N^+^–CH_2_ and –N^+^–CHR groups appeared between δ = 3.83 and 5.4 ppm and confirmed the union of the alkylating agent with tetrandrine. The chemical shifts for the 10 aromatic protons of the tetrandrine appear at δ = 6.30 (s, 1H), 6.76 (dd, *J* = 9.5, 2.1 Hz, 1H), 6.76 (d, *J* = 2.3 Hz, 1H), 6.79 (d, *J* = 2.8 Hz, 2H), 6.82 (dd, *J* = 8.2, 2.6 Hz, 1H), 6.87 (s, 1H), 6.99 (dd, *J* = 8.4, 2.7 Hz, 2H), 7.17 (dd, *J* = 8.2, 2.0 Hz, 1H) and 7.51 (dd, *J* = 8.5, 2.1 Hz, 1H). Finally, signals for the 12 hydrogens of quinoline were found between 7.6 and 8.7 ppm. MS-APCI­(+) *m*/*z*: 986.8 ([DQT–Br]^+^, 23.40%), 453.8 ([DQT–2Br]^2+^, 100%). MS (ESI^+^) *m*/*z*: 987.2 ([DQT–Br]^+^, 30%), 453.2 ([DQT–2Br]^2+^, 100%). Anal. Calcd for C_58_H_58_Br_2_N_4_O_6_·3.5H_2_O: C, 61.65; H, 5.80; N, 4.96. Found: C, 61.63; H, 5.70; N, 4.95.

#### 4.3.2 Synthesis of **DAcT**

It was prepared according to the general procedure using 2-bromo-2′-acetonaphthone (181 mg, 0.72 mmol) under reflux for 24 h. Recrystallization from water/acetone afforded the product as an off-white powder (238 mg, 73%). Decomposition point: 180.1–184.1 °C. UV–vis (DMSO/phosphate buffer, 1:9 v/v), λ_max_ (nm), ε (dm^3^ mol^–1^ cm^–1^): 340, 3875. ^1^H NMR (DMSO-*d*_6_): partial signal assignment was supported by two-dimensional NMR techniques. Signals for –N^+^–CH_3_ and –O–CH_3_ were found at δ = 2.61 (s, 3H), 2.66 (s, 3H), 3.2 (s, 3H), 3.59 (s, 3H), 3.72 (s, 3H), 3.88 (s, 3H). Signals for –N^+^–CH_2_ and –N^+^–CHR groups appear between δ = 3.97 and 5.8 ppm and confirm the union of the alkylating agent with tetrandrine. The chemical shifts for nine of the ten aromatic protons of the tetrandrine skeleton are at δ = 6.01 (s, 1H), 6.57 (dd, *J* = 8.3, 2.2 Hz, 1H), 6.67 (d, *J* = 1.9 Hz, 2H), 6.79 (d, *J* = 1.9 Hz, 1H), 7.05 (dd, *J* = 8.2, 2.7 Hz, 1H), 7.09 (d, *J* = 8.4 Hz, 1H), 7.12 (dd, *J* = 8.3, 2.7 Hz, 1H), 7.23 (dd, *J* = 8.3, 2.0 Hz, 1H). Finally, signals for the 14 hydrogens of naphthalene units appeared between the range 7.6 and 8.75 ppm. MS-APCI­(+) *m*/*z*: 1040.0 ([DAcT–Br]^+^, 13.95%), 480.2 ([DAcT–2Br]^2+^, 100%). MS-ESI­(+) *m*/*z*: 1041.1 ([DAcT–Br]^+^, 100%), 480.9 ([DAcT–2Br]^2+^, ca. 87%). Anal. Calcd for C_62_H_60_Br_2_N_2_O_8_·3.5H_2_O: C, 62.89; H, 5.70; N, 2.37. Found: C, 62.76; H, 5.46; N, 2.50.

#### 4.3.3 Synthesis of **DeBT**

**DeBT** was prepared according to the general procedure using (2-bromoethyl)­benzene (163 mg, 0.88 mmol) under reflux for 72 h. After removal of the solvent under reduced pressure, ethyl acetate was added to induce precipitation. The resulting solid was collected by filtration, washed with ethyl acetate, and recrystallized from water/acetone to afford the product as a white powder (115 mg, 40%). Decomposition point: 213.6–216.7 °C. UV–vis (DMSO/phosphate buffer, 1:9 v/v), λ_max_ (nm), ε (dm^3^ mol^–1^ cm^–1^): 280, 6949. ^1^H NMR (DMSO-*d*_6_): partial signal assignment was supported by two-dimensional NMR techniques. The signals corresponding to the –N^+^–CH_3_ and –O–CH_3_ groups were observed at δ = 2.51 (s, 3H), 2.89 (s, 3H), 3.41 (s, 3H), 3.53 (s, 3H), 3.85 (s, 3H). The resonances assigned to the –N^+^–CH_2_ and –N^+^–CHR fragments appeared between 4.0 and 5.20 ppm. The aromatic protons of the tetrandrine skeleton resonated within the range 6.25–7.1 ppm, partially overlapping with the signals corresponding to the benzyl aromatic protons, which appeared as two multiplets centered at 7.19 and 7.29 ppm. MS-APCI­(+) *m*/*z*: 416.2 ([DeBT–2Br]^2+^, 21.86%). MS-ESI­(+) *m*/*z*: 913.5 ([DeBT–Br]^+^, ca. 5%), 416.8 ([DeBT–2Br]^2+^, ca. 4%). Anal. Calcd for C_54_H_60_Br_2_N_2_O_6_·3.5H_2_O: C, 61.42; H, 6.40; N, 2.65. Found: C, 61.50; H, 6.47; N, 2.89.

### 4.4 Inoculum Preparation and Standardization

A 0.5 McFarland turbidity standard was prepared from a mixture of 99.5 mL of 1% (v/v) sulfuric acid and 0.5 mL of 1.175% (w/v) barium chloride in distilled water. The optical density at 600 nm (OD_600_) of appropriate dilutions was measured and used as a reference for inoculum standardization.^26^

For inoculum preparation, colonies from fresh cultures of each bacterial strain were transferred to nutrient broth (BD Bioxon, Mexico) and incubated at 35–37 °C for 18–24 h. The turbidity of the resulting cultures was adjusted to match the 0.5 McFarland standard, corresponding to approximately 1 × 10^8^ CFU/mL.

### 4.5 Antibacterial Activity

The antibacterial activities of the tetrandrine derivatives were evaluated in triplicate against *E. coli* ATCC 25922, *S. aureus* ATCC 25923, *E. faecalis* ATCC 29212, and *P. aeruginosa* ATCC 10145 obtained from the ATCC. Antibacterial susceptibility testing was performed using a modified broth microdilution assay based on the Clinical and Laboratory Standards Institute (CLSI) guidelines.^15^

Stock solutions of the compounds were prepared in DMSO and diluted with nutrient broth. Aliquots containing the desired concentrations were dispensed into sterile microtiter plates and mixed with standardized bacterial suspensions. Final compound concentrations ranged from 6.25 to 200 μg/mL.

Nutrient broth, bacterial inoculum, DMSO vehicle, ampicillin, and chloramphenicol were included as controls. Plates were incubated at 37 °C for 24 h, and bacterial growth was determined by optical density at 600 nm (OD_600_) using a BioTek 800 TS microplate reader. Subsequently, resazurin solution (15 μL, 0.02%) was added to each well, and the plates were incubated for an additional 4 h at 37 °C. Photographs of the plates were then recorded. MIC and IC_50_ values were estimated by Probit survival/reliability analysis using NCSS 10 software.^44^

### 4.6 Antioxidant Activity

The antioxidant activities of **DBT**, **DeBT**, **DIT**, **DNT**, **DQT**, **DAcT**, and **DAT** were evaluated using DPPH assay and ABTS assay.

#### 4.6.1 DPPH^•^ Assay

The DPPH radical scavenging activity was determined according to a previously reported method.^45^ Samples were dissolved in methanol. An aliquot (20 μL) of each compound stock solution was mixed with 200 μL of DPPH^•^ solution (0.004% w/v) to obtain a final compound concentration of 30 μg/mL. The reaction mixtures were incubated in the dark for 30 min at 25 °C, and the absorbance was measured at 517 nm using a Thermo Fisher Scientific Multiskan GO microplate reader. Radical scavenging activity was expressed as percentage inhibition according to eq 1. Methanol was used as a negative control and gallic acid as a positive control.

1 $$\%\;inhibition=\frac{A_{control}-A_{sample}}{A_{control}}\times 100$$

where *A*_control_ is the absorbance of the DPPH solution without the sample and *A*_sample_ is the absorbance in the presence of the compound.

#### 4.6.2 ABTS^•+^ Assay

The ABTS radical cation scavenging activity was evaluated using a method based on the decolorization of the ABTS^•+^ radical.^46^ The ABTS^•+^ solution was prepared by dissolving 19.3 mg of ABTS in 5 mL of water and allowing the mixture to stand in the dark at 25 °C for 16 h prior to use. The resulting solution was diluted with ethanol to obtain an appropriate absorbance. Aliquots (20 μL) of compound stock solutions were added to 270 μL of the ABTS^•+^ solution to obtain final compound concentrations of 1–30 μg/mL. After incubation for 30 min at room temperature, the absorbance was measured at 734 nm.

Results were expressed as the percentage inhibition of the radical, and IC_50_ values were determined for **DeBT**. Methanol and gallic acid were used as the negative and positive controls, respectively.

#### 4.6.3 Cytotoxicity Assay

Cytotoxicity was evaluated using the (3-(4,5-dimethylthiazol-2-yl)-2,5-diphenyltetrazolium bromide) (MTT) reduction assay with minor modifications according to a previously reported methodology.^47^ ARPE-19, MCF-7, HeLa, and A549 cells were seeded in 96-well plates at a density of 1 × 10^4^ cells per well in 50 μL of culture medium and incubated for 24 h at 37 °C under a humidified atmosphere containing 5% CO_2_. Subsequently, 50 μL aliquots of Dulbecco’s modified Eagle medium supplemented with 5% fetal bovine serum containing different concentrations of the compounds (maximum concentration: 200 μg/mL), previously dissolved in DMSO, were added to each well, and the plates were incubated for an additional 48 h. The maximum DMSO concentration did not exceed 2% (v/v). During the last 4 h of incubation, the medium was replaced with fresh medium containing MTT solution (5 mg/mL, 10 μL/well). The resulting formazan crystals were dissolved with 100 μL of acidified isopropanol, and absorbance was measured at 570 and 630 nm using a Multiskan GO microplate spectrophotometer (Thermo Fisher Scientific Inc., Waltham, MA). Cells treated only with DMSO were considered as the 100% viability control. Cytotoxicity was expressed as IC_50_ values.

### 4.7 DNA Binding

The DNA binding abilities of the tetrandrine derivatives were evaluated using the ethidium bromide (EtBr) displacement assay. This competitive method is based on the decrease in fluorescence intensity of the ethidium bromide–DNA complex upon addition of a competing ligand.^23^ The quenching constants (*K*_sv_) for tetrandrine (**TET**), **DBT**, **DIT**, **DNT**, and **DAT** were previously reported.^25^ In the present work, the quenching constants for **DQT**, **DAcT**, and **DeBT** were determined.

For these measurements, increasing concentrations of each tetrandrine derivative were added to a solution of the dsDNA–EtBr complex ([DNA] = 10 μM, [EtBr] = 5 μM). Fluorescence emission spectra were recorded with an excitation wavelength of 510 nm. The interaction of the compounds with the DNA-bound EtBr was evaluated by monitoring the decrease in fluorescence intensity. The quenching constants (*K*_sv_) were obtained using the Stern–Volmer equation (eq 2):^26^

2 $$\frac{I_{0}}{I}=K_{sv}\left[Q\right]+1$$

where *I*_0_ and *I* are the fluorescence intensities of the dsDNA–EtBr complex in the absence and in the presence of quencher (*Q*), respectively, and *K*_sv_ corresponds to the slope of the linear plot of *I*_0_/*I* versus [Q].

### 4.8 Molecular Modeling and Docking Studies

Geometry optimizations were performed using DFT with the composite B97-3c functional,^48^ as implemented in ORCA 6.1.^49^ This method includes D3­(BJ) dispersion corrections^50^ and a triple-ζ basis set (def2-mTZVP),^51^ providing a balanced description of accuracy and computational cost for medium-sized organic systems. The nature of each stationary point was confirmed by frequency calculations, ensuring the absence of imaginary frequencies.

### 4.9 Docking Studies

Geometry-optimized structures of the tetrandrine derivatives obtained at the DFT level were used for docking studies. Por favor, reemplácelo con: Las estructuras cristalinas de las proteínas objetivo, incluyendo la β-lactamasa TEM-1 (*E. coli*, PDB ID: 1ZG4), la β-lactamasa AmpC (*P. aeruginosa*, PDB ID: 4GZB), la proteína de unión a penicilina 4 (PBP4, *E. faecalis*, PDB ID: 6BSQ), la cloranfenicol acetiltransferasa III (*S. aureus*, PDB ID: 3CLA)­y la proteína de unión a penicilina 2a (PBP2a, *S. aureus* resistente a meticilina, PDB ID: 1VQQ) se recuperaron del Protein Data Bank. Protein structures were prepared using the Protein Preparation Wizard implemented in the Schrödinger Suite,^52^ including removal of nonessential molecules, addition of hydrogen atoms, assignment of bond orders, and restrained energy minimization using the OPLS4 force field.^53^

Ligand structures of the tetrandrine derivatives were obtained from the DFT-optimized geometries described above. Reference compounds (ampicillin, avibactam, and chloramphenicol) were constructed and prepared using LigPrep,^52^ considering relevant protonation states at physiological pH (7.4 ± 0.5) and minimizing their energies prior to docking.

Molecular docking calculations were carried out using Glide.^54^ The docking grid was centered on the active site defined by the cocrystallized ligand. Ligands were treated as flexible during docking, and the best-ranked binding poses were selected based on GlideScore values. Binding affinities (Δ*G*, kcal/mol) were estimated using the GlideScore SP scoring function, and protein–ligand interactions were analyzed using BIOVIA Discovery Studio Visualizer.^55^

## Supplementary Material



## Data Availability

The data that support the findings of this study are available in Supporting Information.

## Notes

^1^H NMR and mass spectra of the new tetrandrine derivatives **DQT**, **DAcT**, and **DeBT**; UV–vis absorption and emission spectra; IC_50_ values against bacterial strains; photographs of microplate cultures with resazurin; emission spectra and fitting curves of the EtBr–DNA system in the presence of **DAcT** and **DeBT**; and molecular docking data, including predicted binding energies, key amino acid interactions, and binding mode diagrams for tetrandrine derivatives and bacterial target proteins (PDF)

## References

[1] World Health Organization (WHO) . Antibiotic Resistance. https://www.who.int/news-room/fact-sheets/detail/antibiotic-resistance (accessed April 22, 2026).

[2] Walsh T. R., Gales A. C., Laxminarayan R., Dodd P. C. (2023). Antimicrobial Resistance:
Addressing a Global Threat to Humanity. PLoS
Med..

[3] Chu W., Yang Y., Cai J., Kong H., Bai M., Fu X., Qin S., Zhang E. (2019). Synthesis and Bioactivities of New
Membrane-Active Agents with Aromatic Linker: High Selectivity and
Broad-Spectrum Antibacterial Activity. ACS Infect.
Dis..

[4] Boucher H. W., Talbot G. H., Benjamin D. K., Bradley J., Guidos R. J., Jones R. N., Murray B. E., Bonomo R. A., Gilbert D. (2013). 10 ×
’20 Progress - Development of New Drugs Active against Gram-Negative
Bacilli: An Update from the Infectious Diseases Society of America. Clin. Infect. Dis..

[5] Liang W., Yu Q., Zheng Z., Liu J., Cai Q., Liu S., Lin S. (2022). Design and Synthesis of Phenyl Sulfide-Based Cationic Amphiphiles
as Membrane-Targeting Antimicrobial Agents against Gram-Positive Pathogens. J. Med. Chem..

[6] Epand R. M., Walker C., Epand R. F., Magarvey N. A. (2016). Molecular
Mechanisms
of Membrane Targeting Antibiotics. Biochim.
Biophys. Acta, Biomembr..

[7] Lee H., Lee D. G. (2019). Programmed Cell Death in Bacterial Community: Mechanisms
of Action, Causes and Consequences. J. Microbiol.
Biotechnol..

[8] Cragg G. M., Grothaus P. G., Newman D. J. (2009). Impact of Natural Products on Developing
New Anti-Cancer Agents. Chem. Rev..

[9] Debnath B., Singh W. S., Das M., Goswami S., Singh M. K., Maiti D., Manna K. (2018). Role of Plant
Alkaloids on Human
Health: A Review of Biological Activities. Mater.
Today Chem..

[10] Rajput A., Sharma R., Bharti R. (2022). Pharmacological
Activities and Toxicities
of Alkaloids on Human Health. Mater. Today:
Proc..

[11] Bhagya N., Chandrashekar K. R. (2016). Tetrandrine
- A Molecule of Wide Bioactivity. Phytochemistry.

[12] Bhagya N., Chandrashekar K. R. (2018). Tetrandrine
and Cancer – An Overview on the
Molecular Approach. Biomed. Pharmacother..

[13] Bhagya N., Chandrashekar K. R. (2022). Autophagy
and Cancer: Can Tetrandrine Be a Potent Anticancer
Drug in the near Future?. Biomed. Pharmacother..

[14] Liu J., Wang F., Wang X., Fan S., Li Y., Xu M., Hu H., Liu K., Zheng B., Wang L., Zhang H., Li J., Li W., Zhang W., Hu Z., Cao R., Zhuang X., Wang M., Zhong W. (2023). Antiviral
Effects and Tissue Exposure of Tetrandrine against SARS-CoV-2 Infection
and COVID-19. MedComm.

[15] Lee Y. S., Han S. H., Lee S. H., Kim Y. G., Park C. B., Kang O. H., Keum J. H., Kim S. B., Mun S. H., Seo Y. S., Myung N. Y., Kwon D. Y. (2012). The Mechanism of
Antibacterial Activity of Tetrandrine against Staphylococcus Aureus. Foodborne Pathog. Dis..

[16] Yi K., Liu S., Liu P., Luo X., Zhao J., Yan F., Pan Y., Liu J., Zhai Y., Hu G. (2022). Synergistic Antibacterial
Activity of Tetrandrine Combined with Colistin against MCR-Mediated
Colistin-Resistant Salmonella. Biomed. Pharmacother..

[17] Shafiq M., Yao F., Bilal H., Rahman S. U., Zeng M., Ali I., Zeng Y., Li X., Yuan Y., Jiao X. (2022). Synergistic
activity of tetrandrine and colistin against *mcr-1*-harboring *Escherichia coli*. Antibiotics.

[18] Lara K. O., Godoy-Alcántar C., Rivera I. L., Eliseev A. V., Yatsimirsky A. K. (2001). Complexation
of Dicarboxylates and Phosphates by a
Semisynthetic Alkaloid-Based Cyclophane in Water. J. Phys. Org. Chem..

[19] Lara K. O., Godoy-Alcántar C., Eliseev A. V., Yatsimirsky A. K. (2004). Recognition
of α-Amino Acid Derivatives by N,N′-Dibenzylated S,S-(+)-Tetrandrine. Org. Biomol. Chem..

[20] Lara K. O., Godoy-Alcántar C., Eliseev A. V., Yatsimirsky A. K. (2005). Ester Cleavage
by *S,S*-(+)-Tetrandrine Bearing Attached Thiol Groups. ARKIVOC.

[21] Moreno-Corral R., Lara K. O. (2008). Complexation Studies of Nucleotides
by Tetrandrine
Derivatives Bearing Anthraquinone and Acridine Groups. Supramol. Chem..

[22] Wong-Molina A., Lara K. O., Sánchez M., Burboa M. G., Gutiérrez-Millán L. E., Marín J. L., Valdez M. A. (2008). Interaction of Calf Thymus DNA with
a Cationic Tetrandrine Derivative at the Air–Water Interface. J. Biomed. Nanotechnol..

[23] Calvillo-Páez V., Sotelo-Mundo R. R., Leyva-Peralta M., Gálvez-Ruiz J.
C., Corona-Martínez D., Moreno-Corral R., Escobar-Picos R., Höpfl H., Juárez-Sánchez O., Lara K. O. (2018). Synthesis, Spectroscopic,
Physicochemical and Structural
Characterization of Tetrandrine-Based Macrocycles Functionalized with
Acridine and Anthracene Groups: DNA Binding and Anti-Proliferative
Activity. Chem. Biol. Interact..

[24] Escobar-Picos R., Vasquez-Ríos M.
G., Sotelo-Mundo R. R., Jancik V., Martínez-Otero D., Calvillo-Páez V., Höpfl H., Ochoa Lara K. (2019). A Chiral Bis-Naphthylated Tetrandrine
Dibromide: Synthesis, Self-Assembly into an Organic Framework Based
on Nanosized Spherical Cages, and Inclusion Studies. ChemPlusChem.

[25] González-Martínez S. M., Valencia-Ochoa D. P., Gálvez-Ruiz J.
C., Leyva-Peralta M. A., Juárez-Sánchez O., Islas-Osuna M. A., Calvillo-Páez V. I., Höpfl H., Íñiguez-Palomares R., Rocha-Alonzo F., Ochoa Lara K. (2022). DNA-Binding Properties of Bis-N-Substituted Tetrandrine
Derivatives. ACS Omega.

[26] Calvillo-Páez V. I., Plascencia-Jatomea M., Ochoa-Terán A., Del-Toro-Sánchez C. L., González-Vega R. I., González-Martínez S. M., Ochoa Lara K. (2023). Tetrandrine
Derivatives as Promising Antibacterial
Agents. ACS Omega.

[27] Gong L., Liu H., Xu B., Yu T., Wang Y., Niu S.-L., Zeng R., Ouyang Q. (2024). Insights on
Exploring the Therapeutic
Potential and Structural Modification of Tetrandrine. Future Med. Chem..

[28] Kadafour A. N. W., Ibrahim H., Bala M. D. (2022). Synthesis, Characterization
and Application
of New Imino-Functionalized 1,3-Diazolium Salts as Antimicrobial Agents. J. Mol. Struct..

[29] Vieira-da-Silva B., Castanho M. A. R. B. (2023). Resazurin
Reduction-Based Assays Revisited: Guidelines
for Accurate Reporting of Relative Differences on Metabolic Status. Molecules.

[30] Njoku D. I., Guo Q., Dai W., Chen J. L., Mao G., Sun Q., Sun H., Peng Y.-K. (2023). The Multipurpose Application of Resazurin in Micro-Analytical
Techniques: Trends from the Microbial, Catalysis and Single Molecule
Detection Assays. TrAC, Trends Anal. Chem..

[31] Zhou Z., Zhou S., Zhang X., Zeng S., Xu Y., Nie W., Zhou Y., Xu T., Chen P. (2023). Quaternary
ammonium
salts: insights into synthesis and new directions in antibacterial
applications. Bioconjugate Chem..

[32] Schaich K. M., Tian X., Xie J. (2015). Hurdles and
Pitfalls in Measuring
Antioxidant Efficacy: A Critical Evaluation of ABTS, DPPH, and ORAC
Assays. J. Funct. Foods.

[33] Prior R. L., Wu X., Schaich K. (2005). Standardized Methods
for the Determination of Antioxidant
Capacity and Phenolics in Foods and Dietary Supplements. J. Agric. Food Chem..

[34] Shirwaikar A., Shirwaikar A., Rajendran K., Punitha I. S. R. (2006). In Vitro Antioxidant
Studies on the Benzyl Tetra Isoquinoline Alkaloid Berberine. Biol. Pharm. Bull..

[35] Brown E. D., Wright G. D. (2016). Antibacterial Drug Discovery in the Resistance Era. Nature.

[36] Xu J., Wang G. A., Gao L., Wu L., Lei Q., Deng H., Li F. (2023). Enabling Programmable Dynamic DNA
Chemistry Using Small-Molecule DNA Binders. Nat. Commun..

[37] Sirajuddin M., Ali S., Badshah A. (2013). Drug–DNA
Interactions and Their Study by UV–Visible,
Fluorescence Spectroscopies and Cyclic Voltammetry. J. Photochem. Photobiol., B.

[38] Kumar K. M., Anbarasu A., Ramaiah S. (2014). Molecular
Docking and Molecular Dynamics
Studies on β-Lactamases and Penicillin Binding Proteins. Mol. BioSyst..

[39] Guan S., Zhong L., Yu H., Wang L., Jin Y., Liu J., Xiang H., Yu H., Wang L., Wang D. (2022). Molecular
Docking and Proteomics Reveals the Synergistic Antibacterial Mechanism
of Theaflavin with β-Lactam Antibiotics against MRSA. Front. Microbiol..

[40] Ortiz-Vargas K. A., Gutierrez-Aguilar R. U., Avina-Verduzco J. A., Garcia-Gutierrez H. A., Ontiveros-Rodriguez J. C., Herrera-Bucio R., Navarro-Santos P. (2024). Molecular Docking and Dynamics of
a Series of Aza-Heterocyclic
Compounds against Penicillin-Binding Protein 2a of Methicillin-Resistant *Staphylococcus aureus*. Chem. Proc..

[41] Bush K., Bradford P. A. (2019). Interplay between β-lactamases
and new β-lactamase
inhibitors. Nat. Rev. Microbiol..

[42] Hartman B. J., Tomasz A. (1984). Low-Affinity Penicillin-Binding Protein
Associated
with β-Lactam Resistance in *Staphylococcus aureus*. J. Bacteriol..

[43] Lionta E., Spyrou G., Vassilatis D. K., Cournia Z. (2014). Structure-Based Virtual
Screening for Drug Discovery: Principles, Applications and Recent
Advances. Curr. Top. Med. Chem..

[44] Prakash G., Boopathy M., Selvam R., Johnsanthosh Kumar S., Subramanian K. (2018). The Effect of Anthracene-Based Chalcone
Derivatives
in the Resazurin Dye Reduction Assay Mechanisms for the Investigation
of Gram-Positive and Gram-Negative Bacterial and Fungal Infection. New J. Chem..

[45] Molyneux P. (2004). The Use of
the Stable Radical Diphenylpicrylhydrazyl (DPPH) for Estimating Antioxidant
Activity. J. Sci. Technol..

[46] Re R., Pellegrini N., Proteggente A., Pannala A., Yang M., Rice-Evans C. (1999). Antioxidant
activity applying an improved ABTS radical
cation decolorization assay. Free Radical Biol.
Med..

[47] Rascón-Valenzuela L., Velázquez C., Garibay-Escobar A., Medina-Juárez L. A., Vilegas W., Robles-Zepeda R. E. (2015). Antiproliferative Activity of Cardenolide
Glycosides from *Asclepias subulata*. J. Ethnopharmacol..

[48] Brandenburg J. G., Bannwarth C., Hansen A., Grimme S. (2018). B97–3c: a revised
low-cost variant of density functional theory. J. Chem. Phys..

[49] Neese F. (2025). Software update:
the ORCA program system—version 6.1. Wiley Interdiscip. Rev.: Comput. Mol. Sci..

[50] Grimme S., Ehrlich S., Goerigk L. (2011). Effect of the damping function in
dispersion-corrected density functional theory. J. Comput. Chem..

[51] Weigend F., Ahlrichs R. (2005). Balanced basis sets
of split valence, triple zeta valence
and quadruple zeta valence quality for H to Rn. Phys. Chem. Chem. Phys..

[52] Schrödinger Release 2023-4; Schrödinger, LLC: New York, NY, 2023.

[53] Lu C., Wu C., Ghoreishi D., Chen W., Wang L., Damm W., Ross G. A., Dahlgren M. K., Russell E., Von Bargen C. D. (2021). OPLS4: improving force field accuracy on challenging regimes of chemical
space. J. Chem. Theory Comput..

[54] Friesner R. A., Banks J. L., Murphy R. B., Halgren T. A., Klicic J. J., Mainz D. T., Repasky M. P., Knoll E. H., Shelley M., Perry J. K. (2004). Glide: a new approach
for rapid, accurate docking
and scoring. J. Med. Chem..

[55] BIOVIA, Dassault Systèmes Discovery Studio Visualizer, Version 2023; Dassault Systèmes: San Diego, CA, USA, 2023.

