# Supplementary material for: N,N′‑Disubstituted Tetrandrine Derivatives: Enhanced Antibacterial Activity via Double Quaternization
Source: ACS Omega. 2026 Aug 4;11(32):48472–82. doi: 10.1021/acsomega.6c05294 (PMC13491889; doi:10.1021/acsomega.6c05294)
Supplement: Supplementary file 1 [file ao6c05294_si_001.pdf]

Supporting Information

N,N′-Disubstituted Tetrandrine Derivatives: Enhanced Antibacterial Activity via Double Quaternization

Viviana I. Calvillo-Páez,[1,2] Adrián Ochoa-Terán,[1] Juan Carlos Gálvez-Ruiz,[3] Martin Samuel Hernández-Zazueta,[2,3] Carmen L. Del-Toro-Sánchez,[4] Luisa A. Rascón-Valenzuela,[3] J. Octavio Juárez-Sánchez,[5] Jancarlo Gomez-Vega,[3] Maribel Plascencia-Jatomea,*[4] and Karen Ochoa Lara*[2]

[1] Centro de Graduados e Investigación en Química, Tecnológico Nacional de México, Campus Tijuana, CP 22444. Tijuana, B. C., México.

[2] Departamento de Investigación en Polímeros y Materiales, Universidad de Sonora, Rosales y Encinas s/n, Col. Centro CP 83000. Hermosillo, Sonora, México. *Corresponding author. E-mail: karen.ochoa@unison.mx

[3] Departamento de Ciencias Químico-Biológicas, Universidad de Sonora, Rosales y Encinas s/n, Col. Centro CP 83000. Hermosillo, Sonora, México.

[4] Departamento de Investigación y Posgrado en Alimentos, Universidad de Sonora, Rosales y Encinas s/n, Col. Centro CP 83000. Hermosillo, Sonora, México. *Corresponding author. E-mail: maribel.plascencia@unison.mx

[5] Departamento de Investigación en Física, Universidad de Sonora, Rosales y Encinas s/n, Col. Centro CP 83000. Hermosillo, Sonora, México.

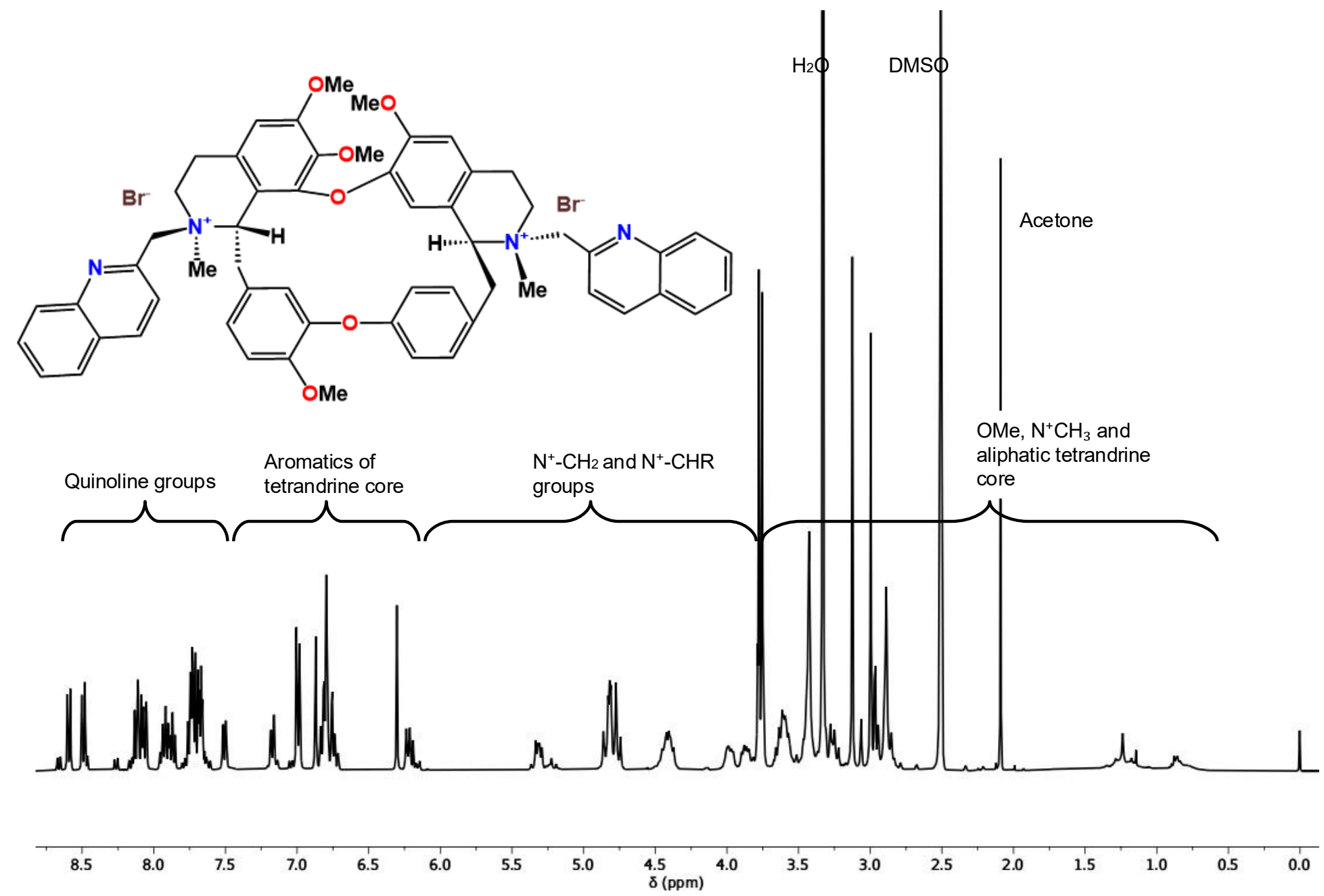

Figure S1. ^{1}H NMR spectrum of **DQT** in DMSO-d_6.

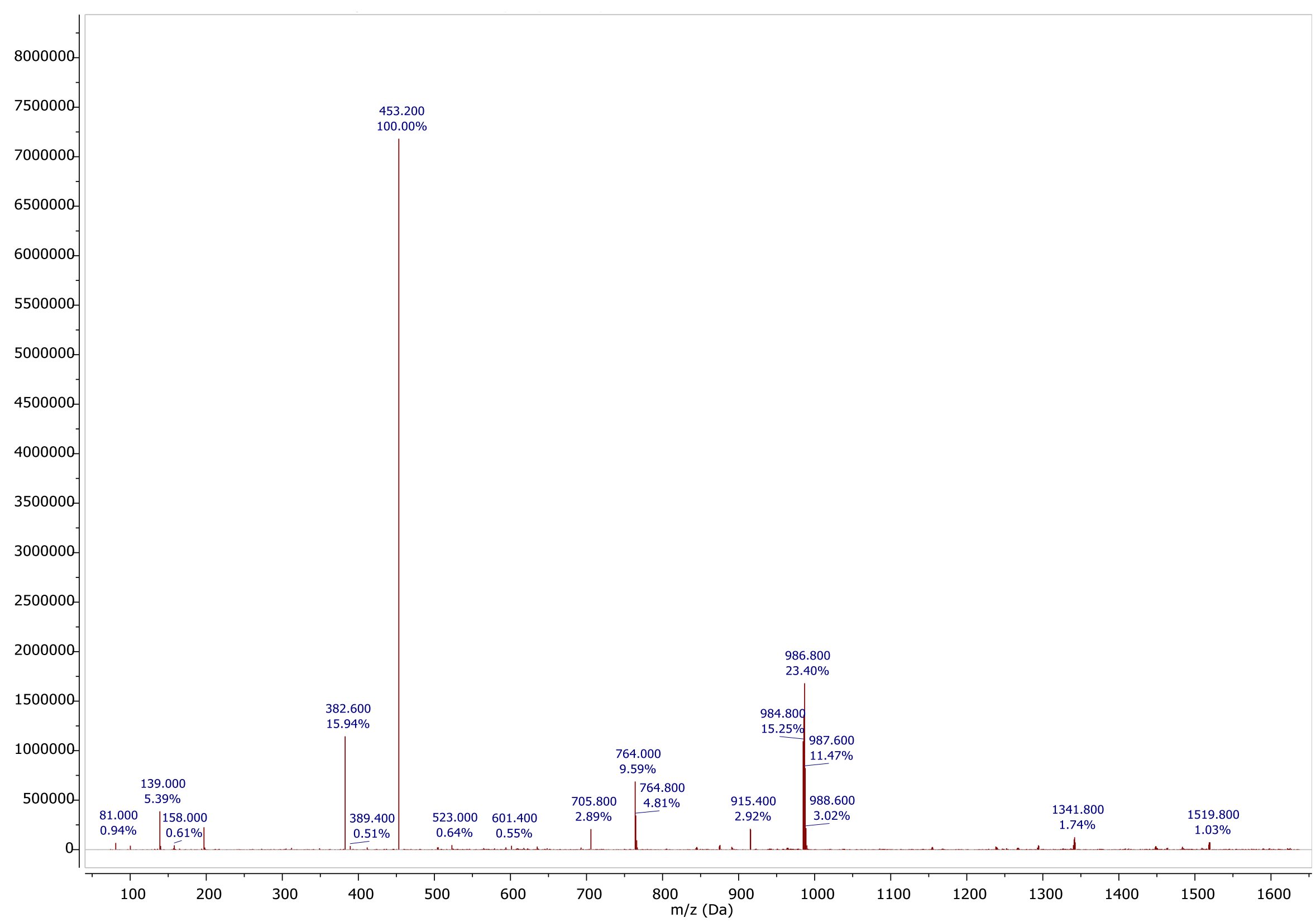

Figure S2. APCI mass spectrum of **DQT**.

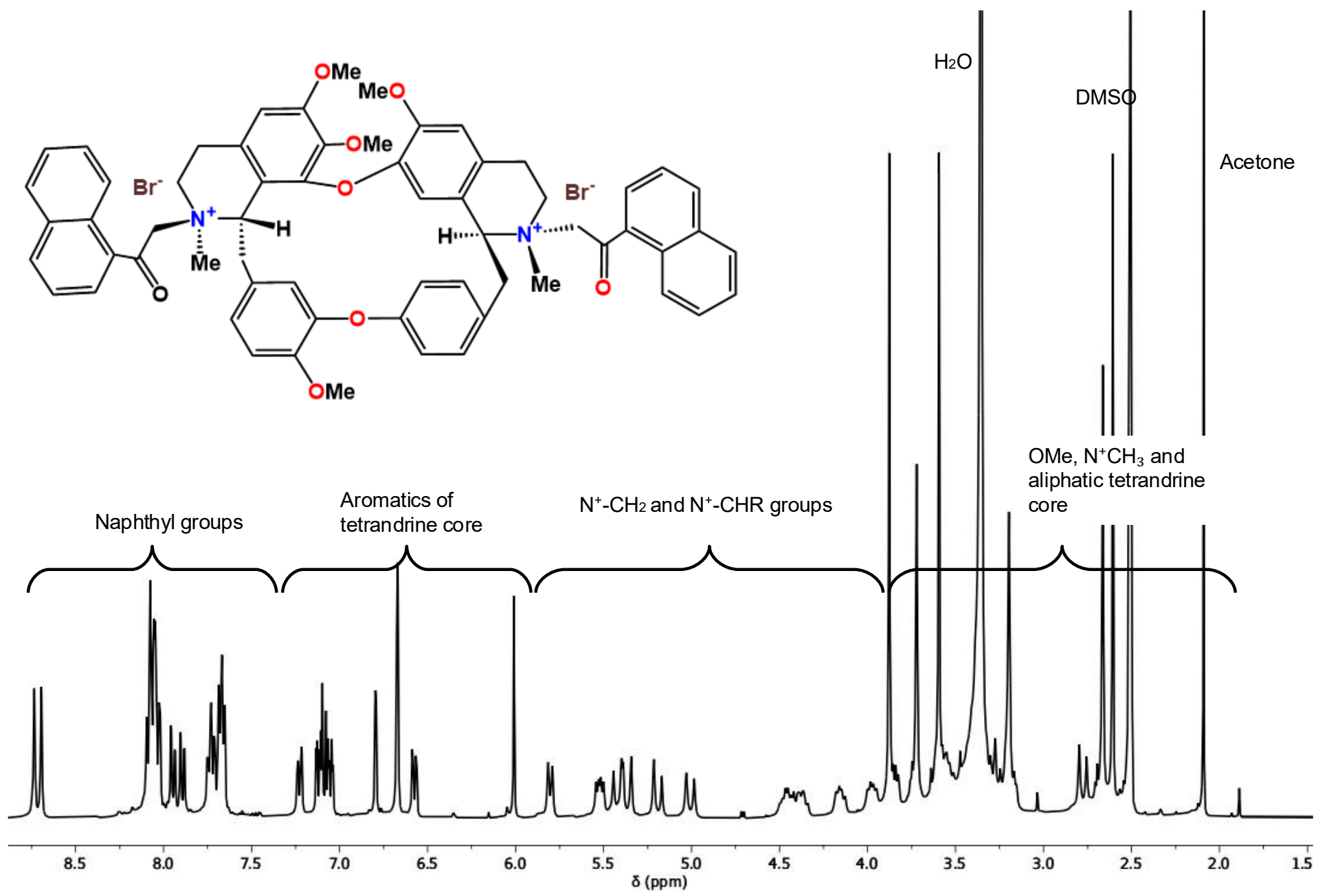

Figure S3. ^{1}H NMR spectrum of **DAcT** in DMSO-d_6.

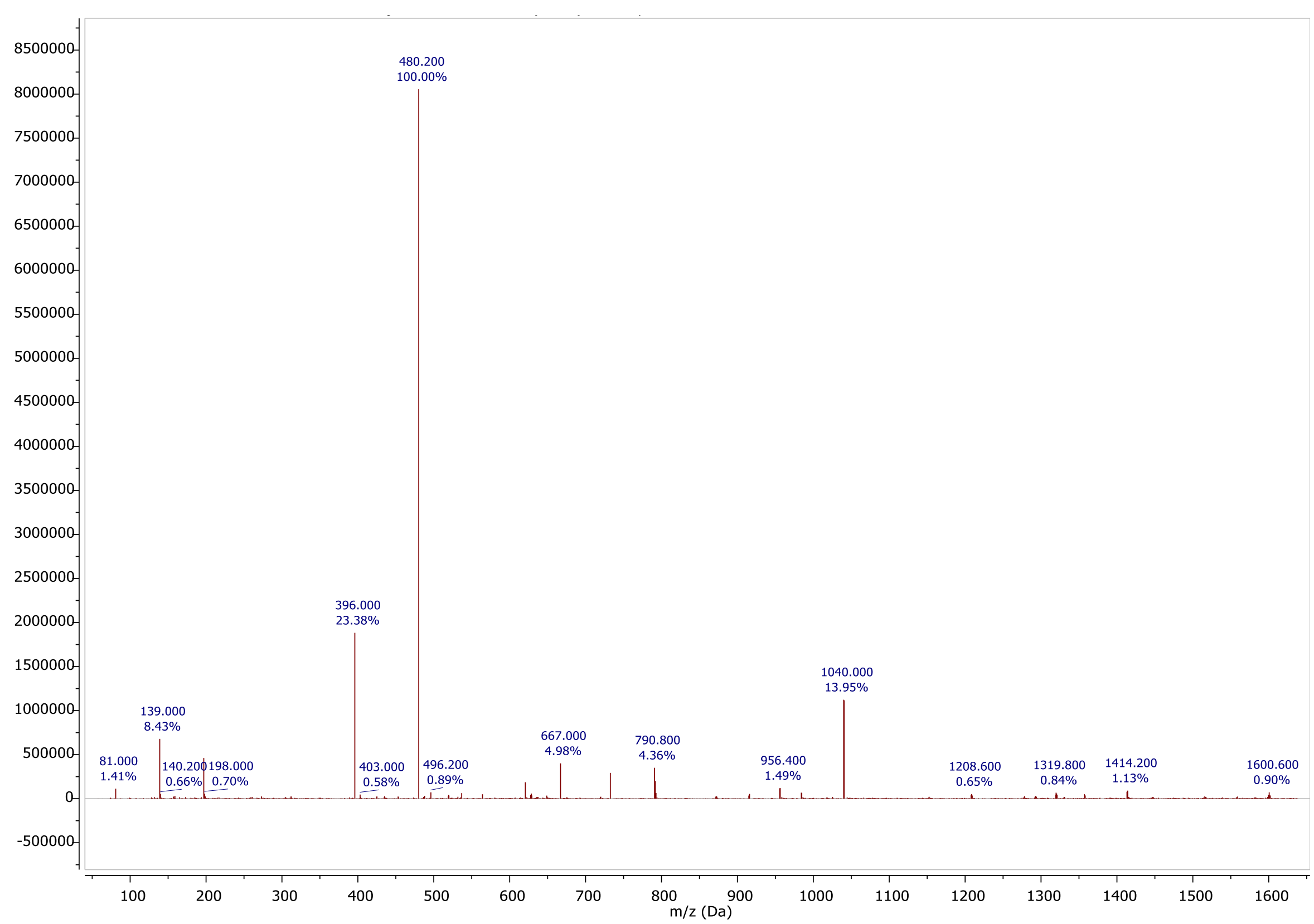

Figure S4. APCI mass spectrum of **DAcT**.

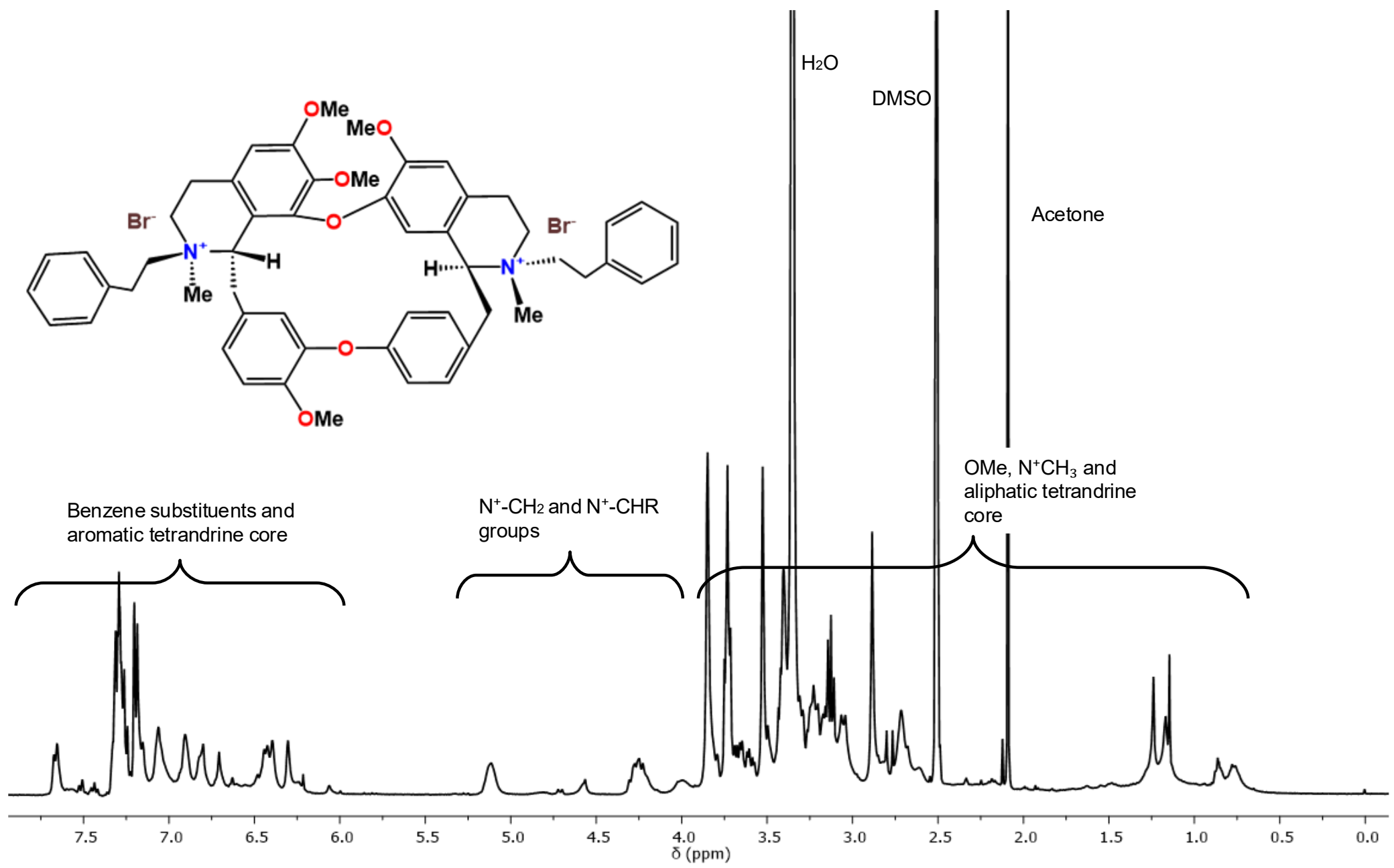

Figure S5. ^{1}H NMR spectrum of **DeBT** in DMSO-d_6.

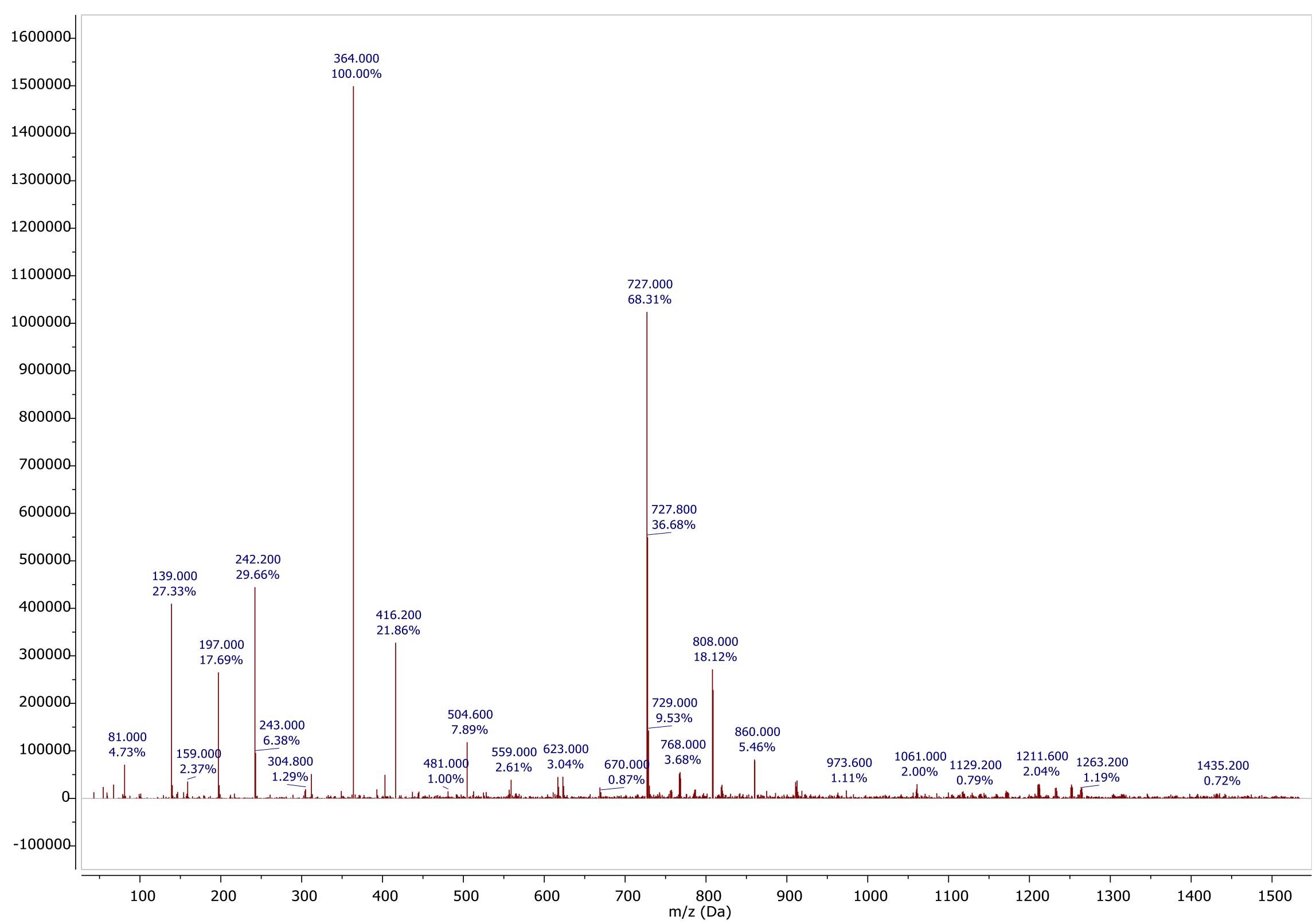

Figure S6. APCI mass spectrum of **DeBT**.

a)

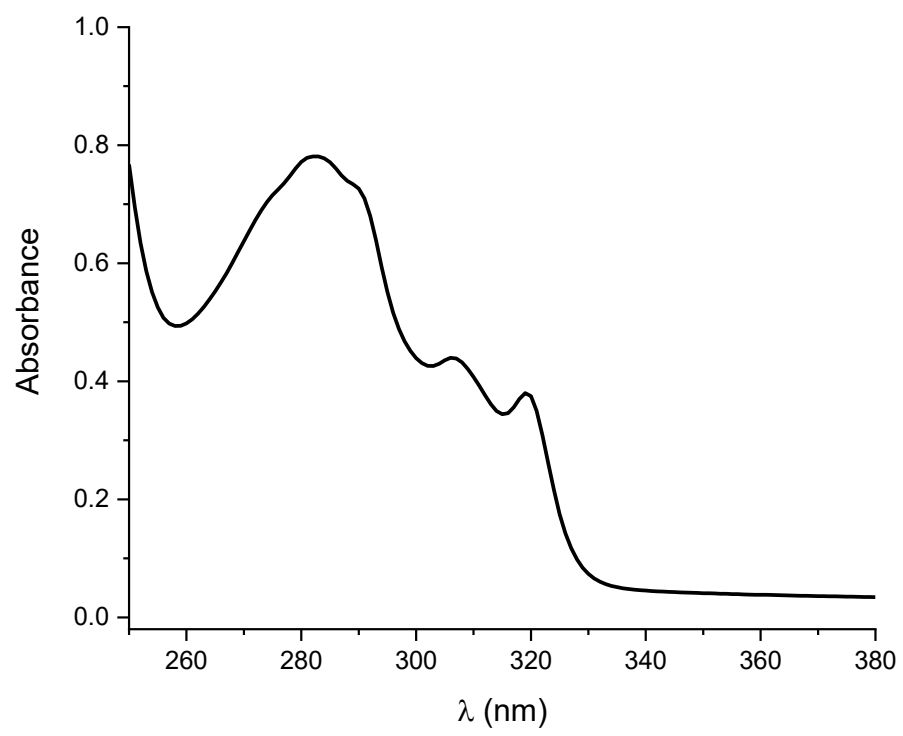

b)

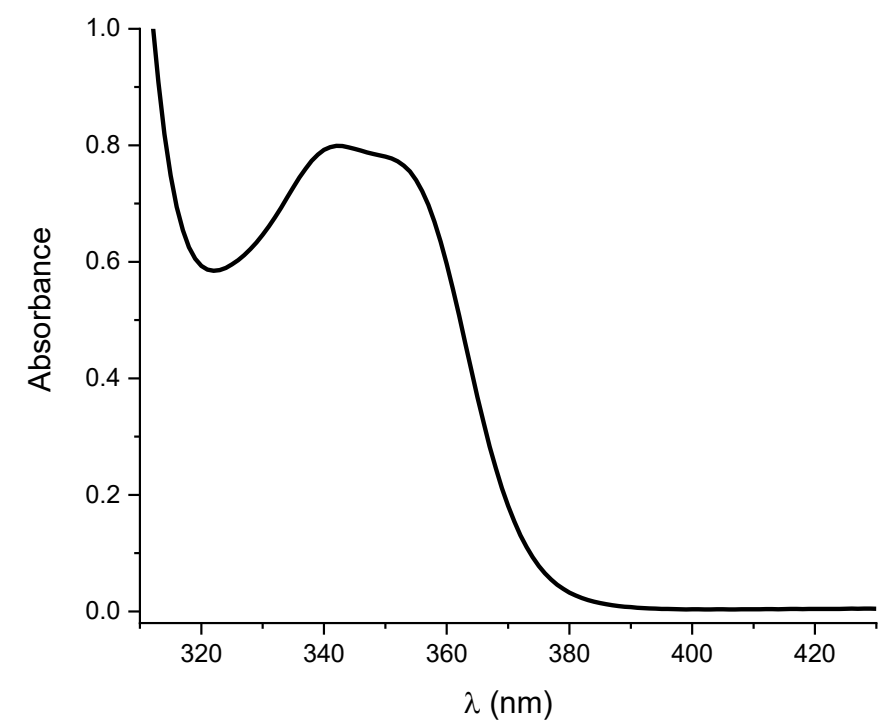

c)

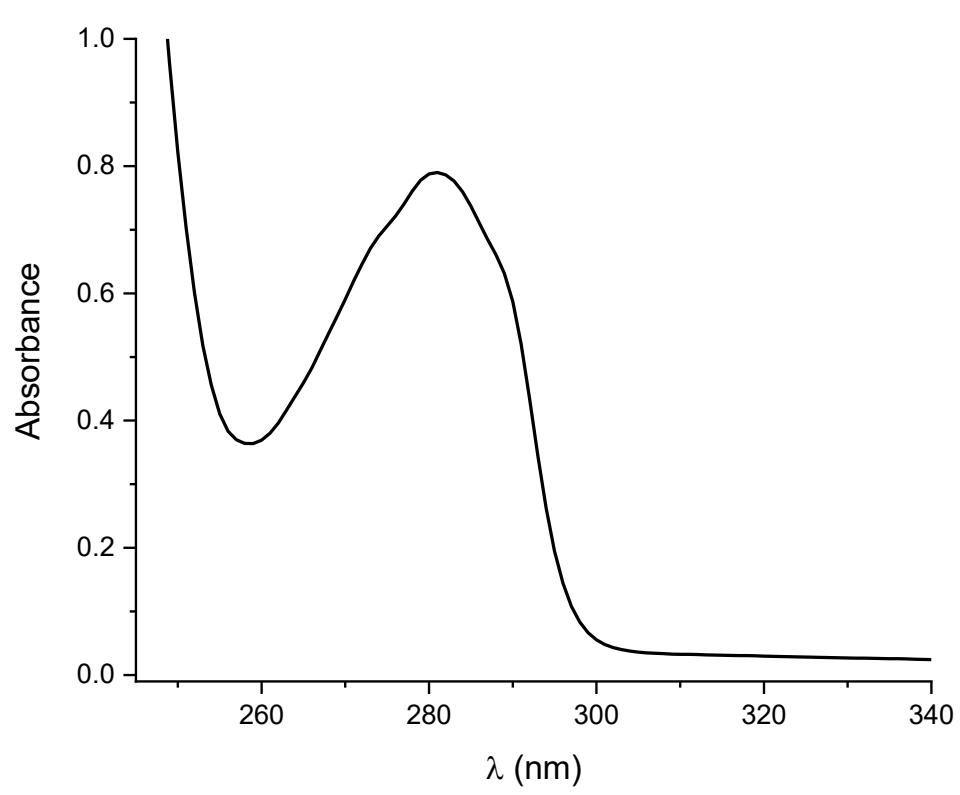

Figure S7. UV-vis absorption spectra of a) **DQT** (7×10^{-5} M), b) **DAcT** (2×10^{-4} M) and c) **DeBT** (1.2×10^{-4} M). All spectra were recorded in phosphate buffer (pH 7.2, 0.01 M NaCl, 10% DMSO (v/v)) at 25 °C.

a)

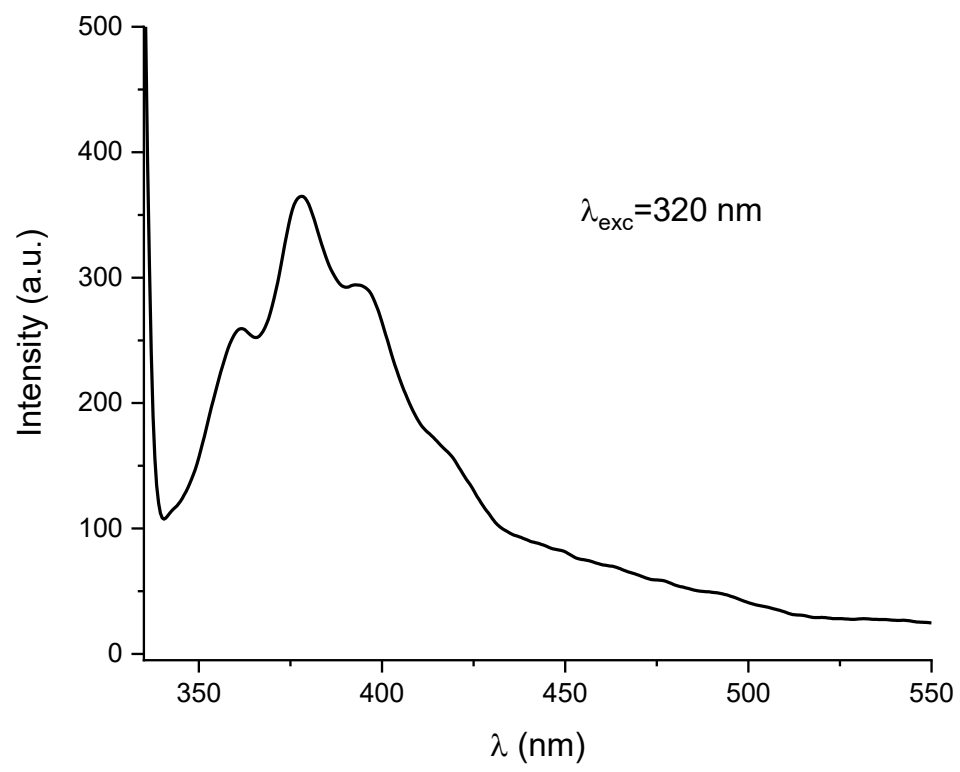

b)

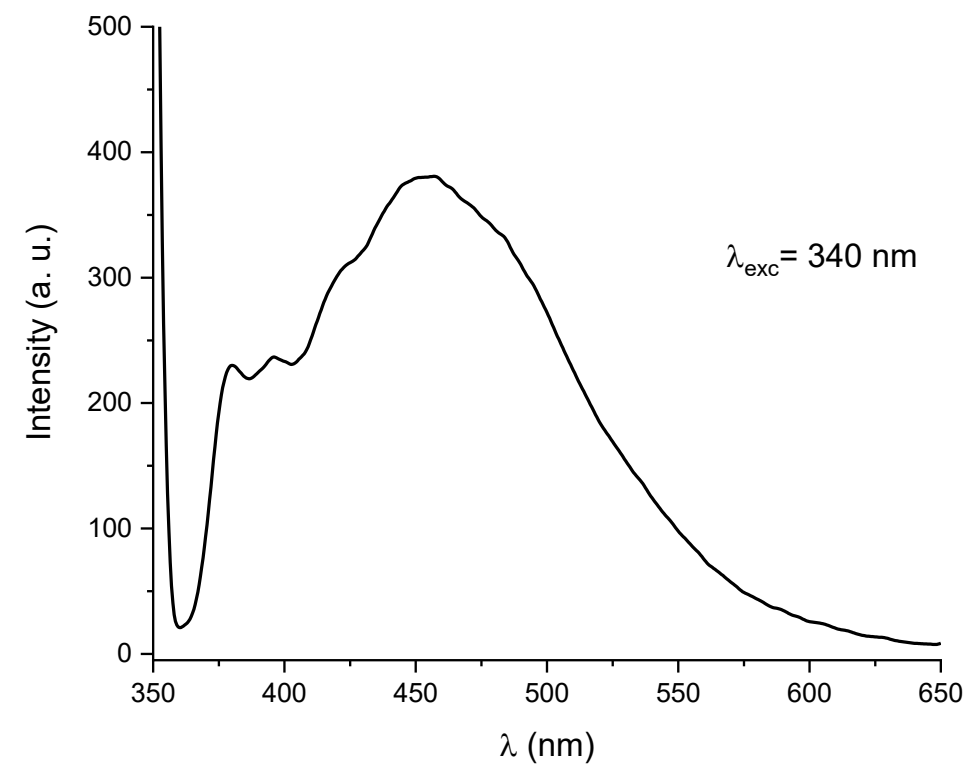

c)

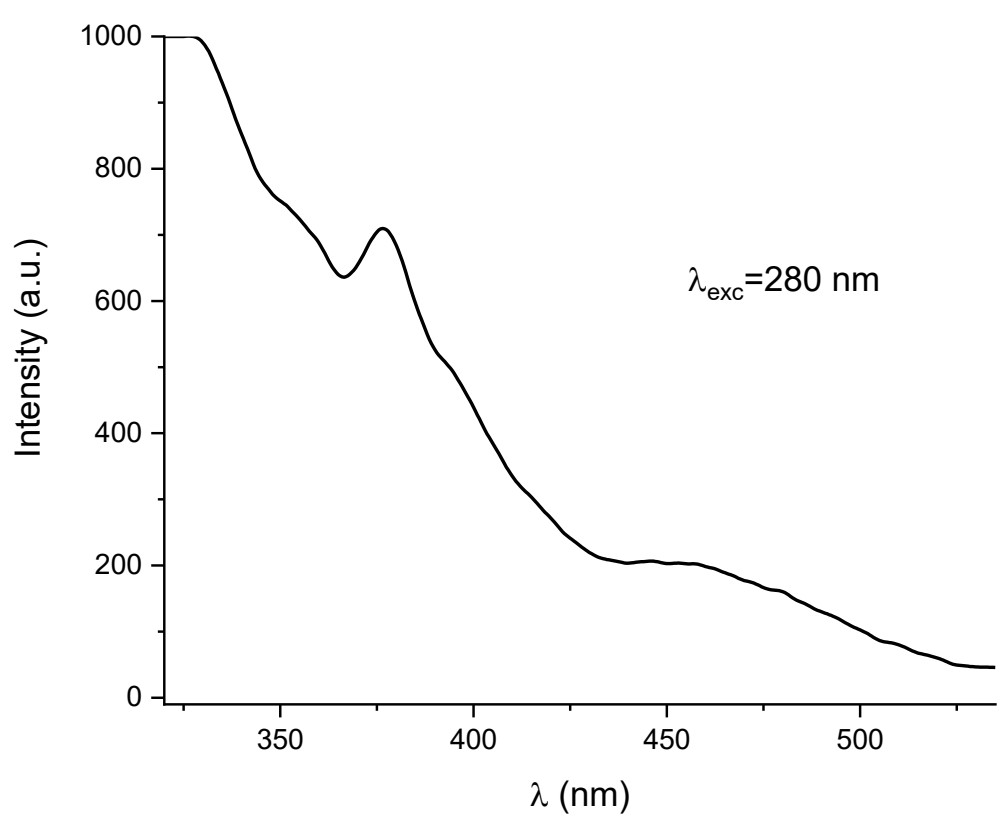

Figure S8. Emission spectra of a) **DQT** (1×10^{-5} M, $\lambda_{exc} = 320$ nm), b) **DAcT** (1×10^{-5} M, $\lambda_{exc} = 340$ nm) and c) **DeBT** (5×10^{-6} M, $\lambda_{exc} = 280$ nm). All spectra were recorded in phosphate buffer (pH 7.2, 0.01 M NaCl, 10% DMSO (v/v)) at 25 °C.

Table S1. Required concentration (µg/mL) of tetrandrine and its derivatives to inhibit the growth of ATCC bacterial strains by 50% (IC_{50}).

	**IC_{50} (µg/mL ± SD)**			
	S. aureus	***E. faecalis***	***E. coli***	***P. aeruginosa***
**DIT**	29.62 ± 0.72	54.15 ± 1.82	ND*	ND*
**DAT**	NI*	NI*	ND*	ND*
**DNT**	1.45 ± 0.16	1.30 ± 0.35	ND*	17.95 ± 0.81
**DBT**	29.14 ± 0.67	13.36 ± 0.27	1.07 ± 0.34	9.22 ± 0.34
**DQT**	2.72 ± 0.41	0.20 ± 0.017	87.38 ± 3.01	100.49 ± 3.67
**DAcT**	2.14 ± 0.40	ND*	27.89 ± 0.94	87.27 ± 4.37
**DeBT**	30.82 ± 1.31	554.14 ± 107.80	516.99 ± 88.05	663.63 ± 168.04
**Tetrandrine**	NI*	NI*	NI*	NI*
**Ampicillin**	1142.97 ± 154.63	56.26 ± 7.06	4.36 ± 0.46	139.54 ± 2.85
**Chloramphenicol**	31.92 ± 1.45	156.28 ± 4.41	6.05 ± 0.28	47.85 ± 1.99

Values are mean ± standard deviation (SD) of three repetitions (n=3). NI*= The IC_{50} value was not obtained at the maximum concentration used. ND*= The variation of the data obtained did not allow the calculation of the IC_{50} with the Probit model used.

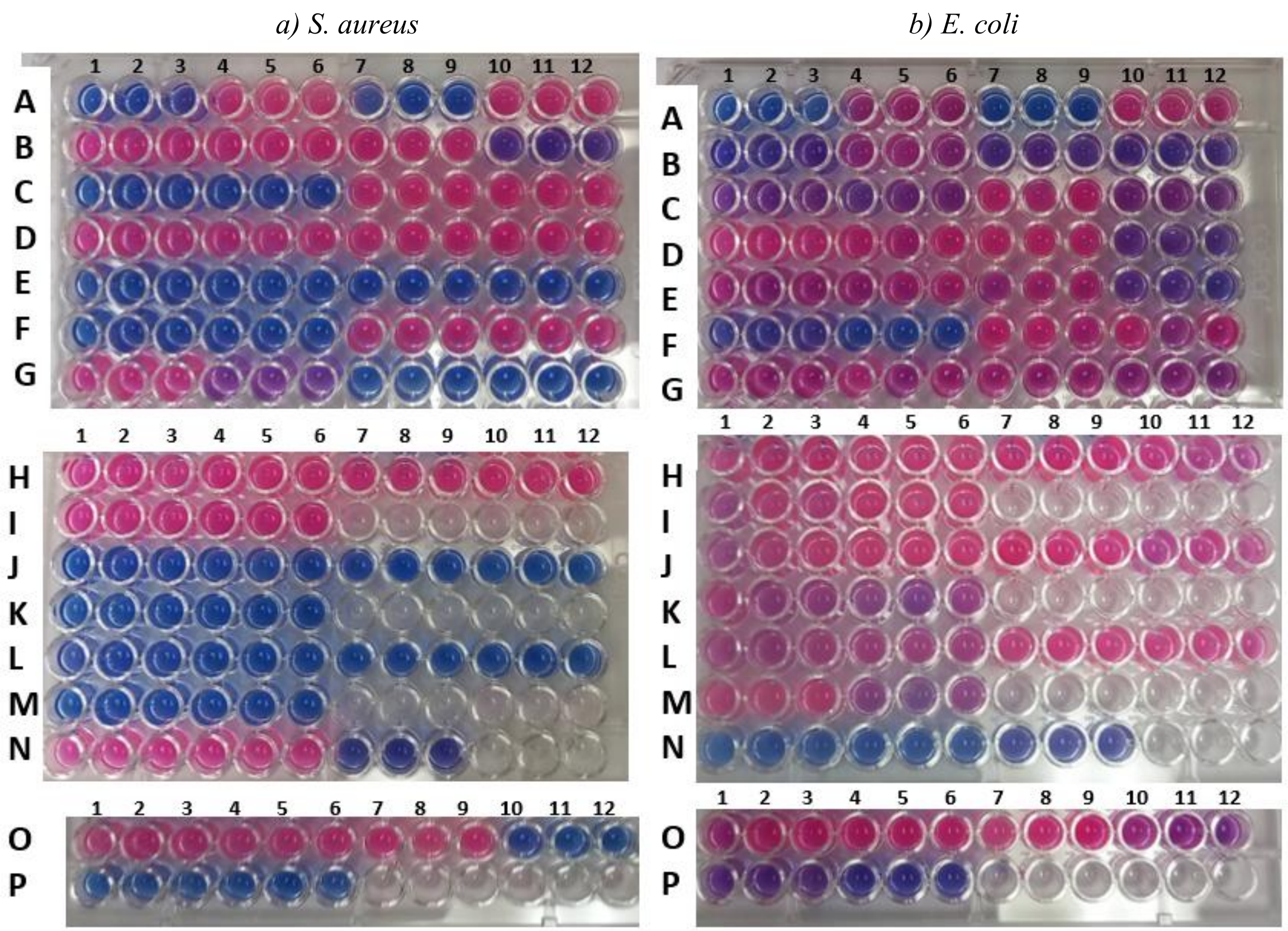
a) *S. aureus*
b) *E. coli*
1 2 3 4 5 6 7 8 9 10 11 12
A
B
C
D
E
F
G
H
I
J
K
L
M
N
O
P

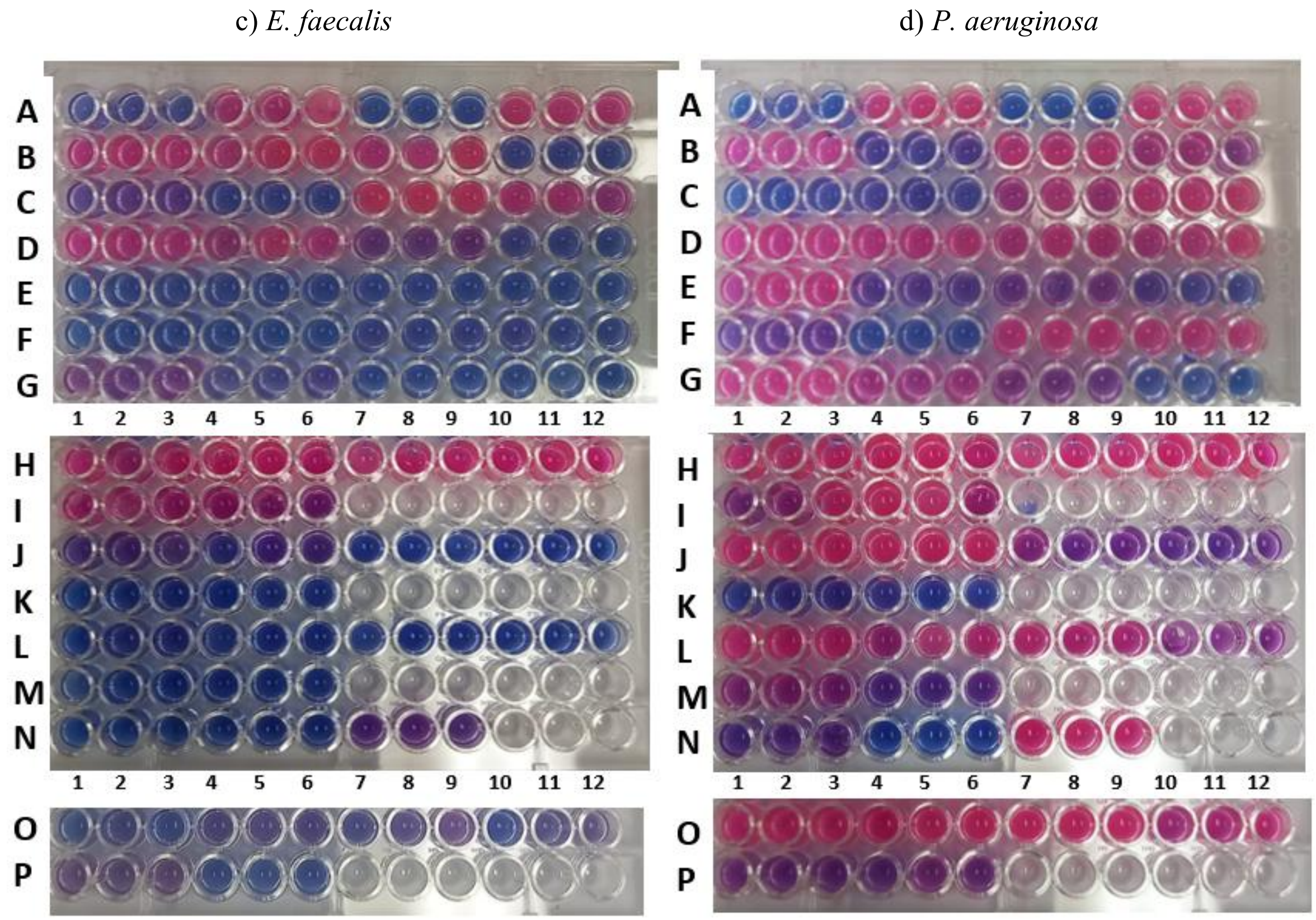

Figure S9. Microplate cultures of the different bacterial strains, with resazurin: a) *S. aureus*, b) *E. coli*, c) *E. faecalis*, and d) *P. aeruginosa*. In all cases: **A1-A3** wells: nutrient broth, **A4-A6:** nutrient broth + respective inoculum, **A7-A9**: nutrient broth + DMSO, **A10-A12:** nutrient broth + respective inoculum + DMSO, **B1-C6: DIT** at 6.25, 12.5, 25, 50, 100 and 200 µg/mL, **C7-D12: DAT** at 6.25, 12.5, 25, 50, 100 and 200 µg/mL, **E1-F6: DNT** at 6.25, 12.5, 25, 50, 100 and 200 µg/mL; **F7-G12**: **DBT** at 6.25, 12.5, 25, 50, 100 and 200 µg/mL; **H1-I6: TET** at 6.25, 12.5, 25, 50, 100 and 200 µg/mL. **J1-K6: DQT** at 6.25, 12.5, 25, 50, 100 and 200 µg/mL. **L1-M6: DAcT** at 6.25, 12.5, 25, 50, 100 and 200 µg/mL. **N1-N3: Ampicillin** 10 µg/mL. **N4-N6:** Ampicillin 30 µg/mL. **N7-N9:** Chloramphenicol 30 µg/mL. **O1-P6**: **DeBT** at 6.25, 12.5, 25, 50, 100 and 200 µg/mL.

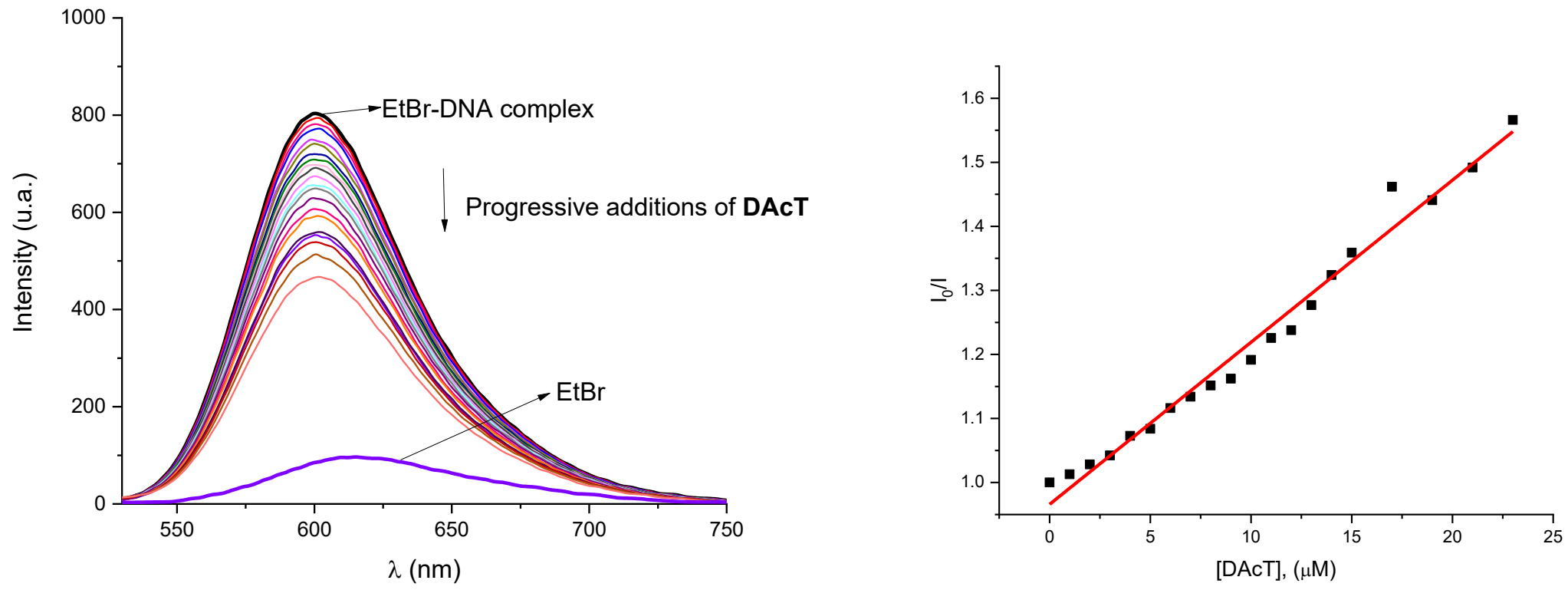

Figure S10. Emission spectra and fitting curve of the EtBr-DNA system in the presence of **DAcT**, λ_{exc} = 510 nm, in phosphate buffer pH = 7.2, 0.01 M NaCl and 10% DMSO (v/v), at 25 °C. [EtBr] = 5 μM, [DNA] = 10 μM, [**DAcT**] = 0-25 μM. DNA: 5′-GTA AGA TGA TTC-3′ and 5′-GAA TCA TCT TAC-3′.

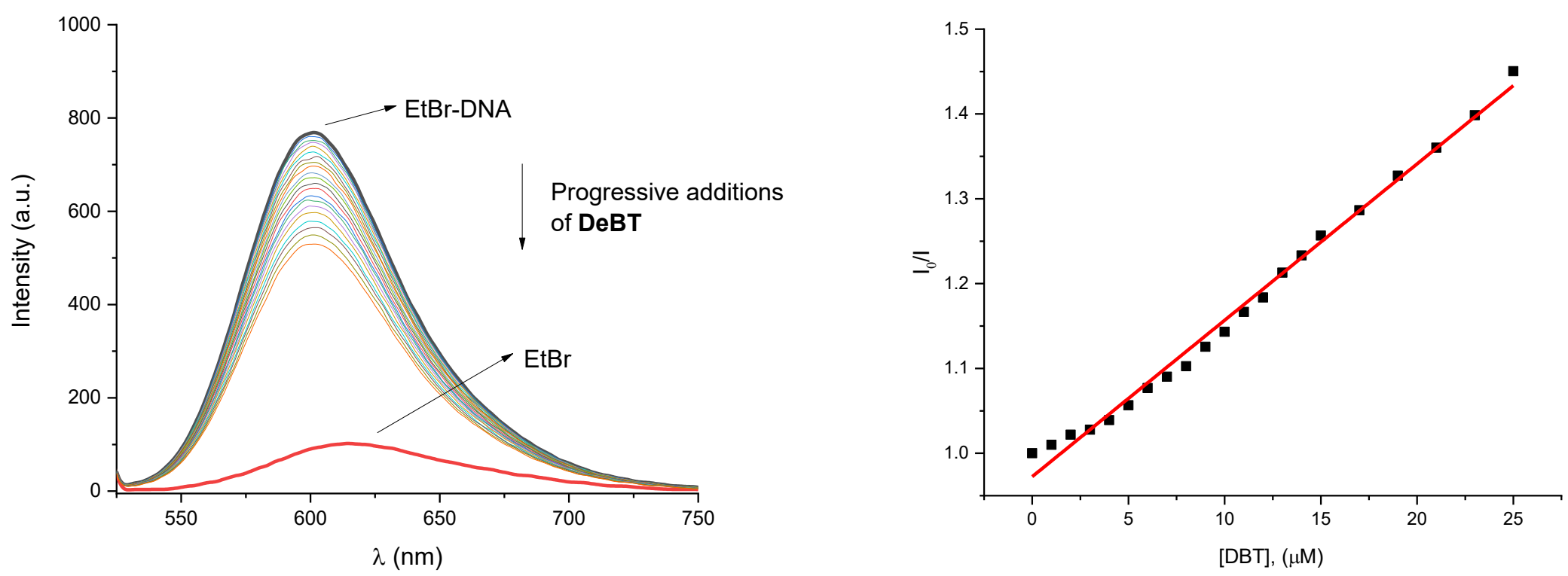

Figure S11. Emission spectra and fitting curve of the EtBr-DNA system in the presence of **DeBT**, λ_{exc} = 510 nm, in phosphate buffer pH = 7.2, 0.01 M NaCl and 10% DMSO (v/v), at 25 °C. [EtBr] = 5 μM, [DNA] = 10 μM, [**DeBT**] = 0-25 μM. DNA: 5′-GTA AGA TGA TTC-3′ and 5′-GAA TCA TCT TAC-3′.

(μM)

Table S2. Binding energy and key amino acid interactions of tetrandrine derivatives and control compounds with bacterial target proteins identified by molecular docking analysis.

Microorganism	Target protein	PDB ID	Compound	CID	Role	MIC (μg/mL ± SD)	ΔG (kcal/mol)	Key residues	Interaction type
Escherichia coli	TEM-1 β-lactamase	1ZG4	Ampicillin	6249	Control	0.018 ± 0.007	-7.0	Ser70, Lys73, Tyr105, Ser130, Asn132, Asn170, Gly104, Pro167, Val216, Gly236, Ala237, Gly238, Lys234, Glu240	H-bond (Ser70, Ser130, Asn132, Gly104, Ala237, Gly236); Carbon H-bond (Pro167, Val216); π-alkyl (Tyr105); Attractive charge (Lys73, Lys234); Unfavorable positive–positive (Lys73)
			DBT	—	Experimental	< 1.07 ± 0.34	-7.7	Tyr105, Pro107, Val216, Lys234, Ser235, Arg244, Gly236, Ala237, Gly238, Asn132, Glu104	π–π stacked (Tyr105); π–σ (Pro107); π-alkyl (Val216, Lys234, Ser235, Arg244); H-bond (Asn132, Gly236); Carbon H-bond (Glu104); van der Waals (Ala237, Gly238)
			Tetrandrine (TET)	73078	TET	NI*	-7.3	Arg258, Tyr260, Pro305, Leu78, Leu85, Asp81, Asp86, Thr79, Arg84, Ala255, Asp254, Gln251	Conventional H-bond (Arg258, Tyr260); Carbon H-bond (Asp81); π-anion (Asp81); π-alkyl / alkyl (Leu78, Leu85, Pro305, Ala255); van der Waals (Thr79, Arg84, Asp86, Asp254, Gln251)
Pseudomonas aeruginosa	AmpC β-lactamase (PDC)	4GZB	Avibactam (co-crystal)	9835049	Control	—	-5.1	Arg234, Gly184, Asp237, His241, Arg244; Leu183, Leu185, Leu245, Ala238	Conventional H-bond (Arg234, Gly184, Asp237, His241, Arg244); Carbon H-bond (Gly184); Alkyl/π-alkyl (Leu183, Leu185, Leu245, Ala238); van der Waals
			DBT	—	Experimental	0.89 ± 0.10	-8.4	Glu309, Thr79, Asp254, Arg84, Leu85, Leu107, Leu78, Tyr260, Arg280, Arg301, Pro305, Arg258, Gln251	Conventional H-bond (Arg258, Gln251); Attractive charge (Arg280, Arg301); π-anion (Asp254); π-alkyl (Leu85, Leu107, Pro305, Tyr260); van der Waals (Glu309, Thr79, Leu78, Arg84)

			Tetrandrine (TET)	73078	TET	NI*	-7.3	Asp81, Leu78, Thr79, Arg84, Leu85, Asp86, Arg258, Tyr260, Pro305, Ala255, Asp254, Gln251	Conventional H-bond (Arg258, Tyr260); Carbon H-bond (Asp81); π-anion (Asp81); π-alkyl / alkyl (Leu78, Leu85, Pro305, Ala255); van der Waals (Thr79, Arg84, Asp86, Asp254, Gln251)
Enterococcus faecalis	PBP4	6BSQ	Ampicillin	6249	Control	< 0.20 ± 0.17	-7.1	Val299, Gly298, Pro242, Thr297, Arg175, Asn189, Arg265, Glu263, Ser264, Thr282, Gln232, Asn394, Trp234	van der Waals (Gly298, Pro242, Thr297, Arg175, Asn189, Arg265, Glu263, Ser264, Thr282, Gln232, Asn394); π–π stacked (Trp234); Alkyl (Val299); unfavorable donor-donor (Trp234)
			DQT	—	Experimental	< 0.20 ± 0.17	-10.5	Val299, Thr297, Pro242, Gly298, Thr282, Asn172, Arg175, Arg265, Glu263, Ser264, Trp234, Phe240, Thr262, Lys260, Asp261	π-σ (Thr297); π-π stacked (Val299, Trp234); π-alkyl/alkyl (Pro242, Phe240, Lys260, Thr262); π-anion (Glu263); van der Waals (Gly298, Thr282, Asn172, Arg175, Arg265, Ser264, Asp261)
			Tetrandrine (TET)	73078	TET	NI*	-8.9	Val299, Thr282, Ser264, Ala393, Ala173, Arg175, Arg265, Glu263, Asn172, Lys174, Asp261, Lys260, Thr262, Pro242, Phe240, Trp234	π-alkyl/alkyl (Val299, Pro242, Phe240, Trp234, Lys260, Thr262); π-σ (Thr282); Carbon H-bond (Glu263); van der Waals (Ser264, Ala393, Ala173, Arg175, Arg265, Asn172, Lys174, Asp261)
Staphylococcus aureus	CAT III	3CLA	Chloramphenicol	5959	Control	1.19 ± 0.15	-6.5	Leu160, Val162, Phe165, Gln192, Val193, Ile172, Tyr168, His194, Gly32, Cys31, Asn159, Asn161	Conventional H-bond (Leu160, Val162, Gln192); Carbon H-bond (Gly32); π-π stacked (Phe165); π-alkyl (Val193, Tyr168); π-donor H-bond (His194); van der Waals (Ile172, Cys31, Asn159, Asn161)
			DAcT	—	Experimental	0.04 ± 0.02	-10.1	Pro459, Ala149, Tyr150, Lys434, Thr439, Met439,	Conventional H-bond (His415, Arg458); Carbon H-bond (Gln358); π-π stacked / π-π T-

					Phe356, Asp356, Gln358, Val359, Val360, Ala361, Thr362, His415, Arg458	shaped (Tyr150, Phe356, Tyr150/459 according to rings); π-alkyl (Pro459, Ala149, Met439, Val359, Val360, Ala361, Thr362); van der Waals (Ala149, Thr439, Lys434)
Tetrandrine (TET)	73078	TET	NI*	-8.7	Tyr56, Pro151, Phe55, Lys54, Phe96, Gln98, Trp152, Met125, Tyr121, Val95, Thr94, Ala149	Carbon H-bond (Phe96, Gln98); π-π stacked (Tyr56, Trp152); π-alkyl (Pro151, Phe55, Lys54, Met125, Tyr121, Val95, Thr94, Ala149); van der Waals

TEM-1 β-lactamase, AmpC β-lactamase (PDC), PBP4, and CAT III are the target proteins evaluated. Δ*G* (kcal/mol) = predicted binding free energies were obtained by molecular docking calculations, lower (more negative) values indicate stronger predicted affinity. MIC (µg/mL) = minimum inhibitory concentration (mean ± SD). CID = PubChem Compound ID. Role: Control = reference drug; TET = tetrandrine; Experimental = tetrandrine derivatives (DBT, DQT, DAcT). NI* = no inhibition detected within the tested range. Key residues (chain A) and interaction types are taken from docking/2D interaction diagrams and classified as: hydrogen bonds (conventional and carbon H-bond), hydrophobic contacts (van der Waals, alkyl/π-alkyl), aromatic interactions (π–π stacking, π–σ), and unfavorable contacts (e.g., donor–donor). Protein structures correspond to the PDB IDs listed in the table.

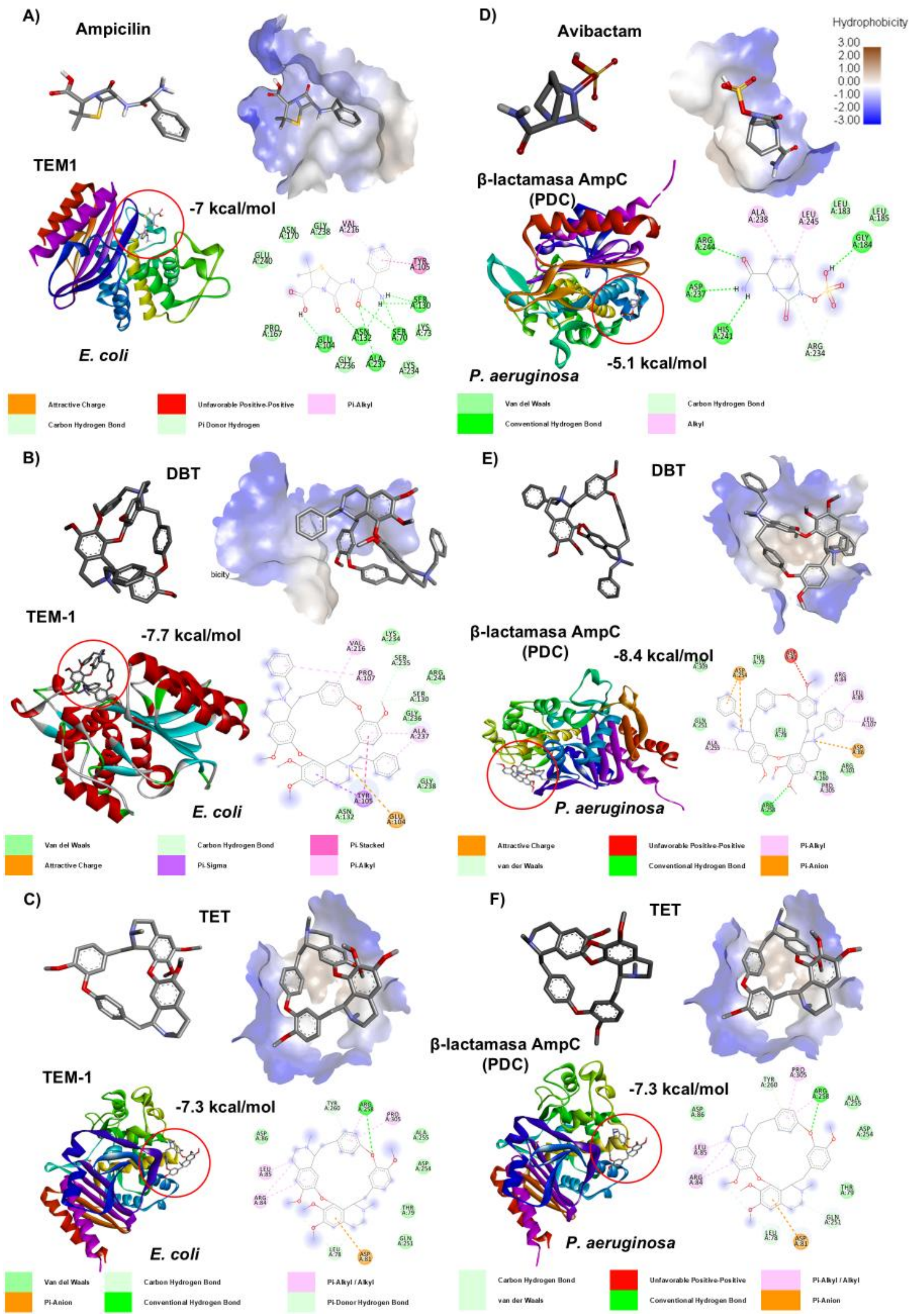

Figure S12. Predicted binding modes and key interactions of reference drugs, **DBT** and **TET** with β-lactamases from Gram-negative bacteria.

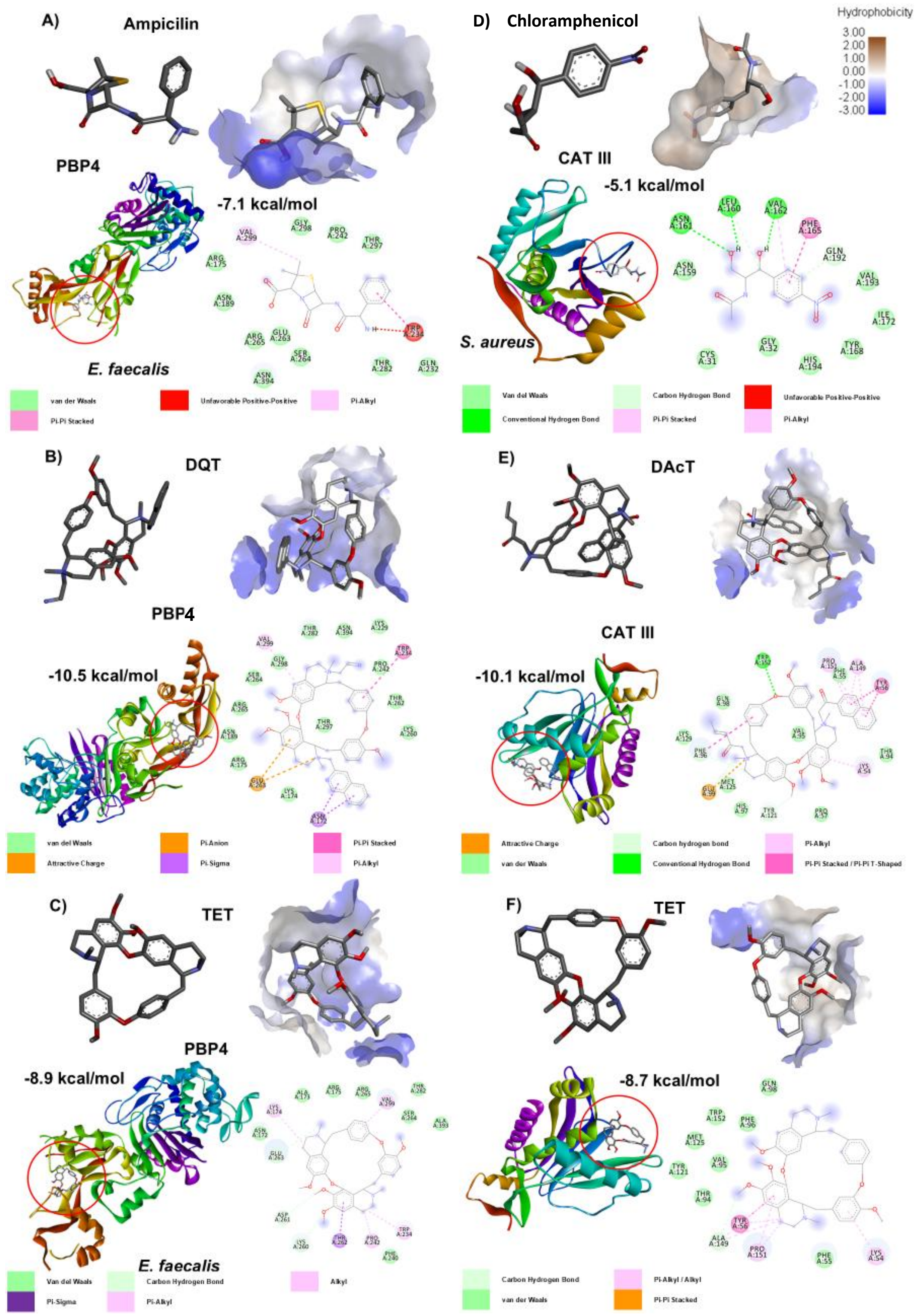

Figure S13. Predicted binding modes and key interactions of reference drugs, **DQT, DAcT** and **TET** with Gram-positive bacterial targets.
